# Long-term trends in juvenile American lobster populations across nine lobster fishing areas in Nova Scotia, Canada

**DOI:** 10.1038/s41598-025-26491-2

**Published:** 2025-11-27

**Authors:** Sitang Arkanit, Henrik Stryhn, K. Fraser Clark, Shannon Scott-Tibbetts, Krishna K. Thakur

**Affiliations:** 1 Department of Health Management, Atlantic Veterinary College, University of Prince Edward Island, Charlottetown, PE C1A 4P3 Canada; 2 Department of Animal Sciences and Aquaculture, Faculty of Agriculture, Dalhousie University, Truro, NS B2N 5E3 Canada; 3 Fishermen and Scientists Research Society, Halifax, NS B3M 4H4 Canada

**Keywords:** Juvenile lobster, Population dynamics, Mixed-effects modelling, Lobster fishing areas, Ecology, Ecology, Ocean sciences

## Abstract

Analyzing juvenile American lobster populations using fishery-independent data enhances understanding of population dynamics and supports fishery sustainability. This repeated cross-sectional study investigated trends in juvenile lobster populations by analyzing size distributions from nine Lobster Fishing Areas (LFAs) in Nova Scotia, Canada. Data were collected in season from lobsters sampled using ventless research traps between 2003 and 2023. Mixed effect linear and logistic regression models assessed spatial and temporal influences on mean carapace length (CL) and proportions of juvenile lobster. This study provides a 20-year overview of mean CL and juvenile probability, accounting for factors including water depth, sampling month, and lobster sex. Results showed significant variation in temporal effects on mean CL and proportion of juvenile lobster between LFAs. Decreasing trends in sampling juvenile lobsters were observed along the Northeastern coast of Cape Breton and in a highly productive area on the South Shore of Nova Scotia. In contrast, other LFAs showed more resilience, with the southwestern area displaying a relatively stable trend. The findings highlight variability in juvenile lobster trends across LFAs, which reflect the relative effectiveness of local management measures and provide insights to inform management decisions in Nova Scotia.

**Supplementary Information:**

The online version contains supplementary material available at 10.1038/s41598-025-26491-2.

## Introduction

The American lobster (*Homarus americanus*) is an important economic resource in Canada. Commercial lobster landings in Canada have been following an increasing trend over the past two decades. In 2022, the annual lobster landings were 98,060 thousand metric tons; accounting for the largest proportion of global lobster catches^1^. Canada exported live, frozen and processed lobsters to the United States, China, EU and other destinations valued at $CAD 3.26 billion in 2021. In the same year, Nova Scotia was the leading exporting province, contributing almost 60% of lobster products exported from major exporting provinces^2^.

Management and conservation strategies, along with legal frameworks, are implemented to address fishing pressure and ensure the sustainability of fisheries in each Lobster Fishing Area (LFA) in Canada^3^. Fishery-dependent stock updates are provided to assess lobster abundance and productivity, as well as to evaluate fishing pressure levels, in order to guide management^4–7^. Several approaches have been developed to analyze lobster stock assessment data; however, methodological complexities—particularly the need to incorporate environmental influences and individual variability—continue to present challenges in accurately predicting lobster recruitment^8,9^. While fishery-dependent data has traditionally played a central role in stock assessments, it may have limitations, such as variability in fishing effort, potential inconsistencies in reporting, and limited spatial coverage that commercial interests can influence^10–12^. These factors are likely to introduce uncertainty and make it difficult to fully capture the dynamics of lobster populations across their life stages, particularly the connection between juvenile recruitment and future harvestable stocks^13^, as the juvenile stage can span 5 to 8 years depending on environmental conditions^14^.

When implemented using robust scientific designs and specialized sampling techniques, fisheries-independent surveys offer a complementary and often more targeted approach for assessing fishery resources, including lobster populations^12^. These surveys are especially valuable for monitoring specific life stages or habitats and have been shown to reduce uncertainty and enhance the reliability of stock assessments^15^. Moreover, they can be cost-effective tools that contribute meaningfully to long-term fisheries management goals^16^. In this study, we integrated environmental variables and individual-level variability into models based on fisheries-independent data. This framework offers a promising approach for future assessments by supporting a more comprehensive understanding of population dynamics and improving the foundation for evidence-based management decisions.

Among several studies on lobster populations, the dynamics of the early-life-stage population are under-discussed due to the limited sampling ability of fishing gear^17^. The requirement for unconventional sampling approaches in juvenile lobster studies have favored experiment-based research or short-term field observations^18^. Most of the previous studies focused primarily on the direct effects of environmental factors on the survivability, growth, development, and behaviors of juveniles^19^, such as temperature^20,21^, water depth^22,23^, habitats, shelter availability^24,25^, predation risk^26,27^, and anthropogenic pressures^28^. However, these well-described factors underlying complex interactions and mechanisms can be challenging to disentangle. We present observations spanning 20 fishing seasons—typically representing 20 years of data for each LFA—using ventless research traps to sample the juvenile lobster population in their natural environments. We explored population trends over this prolonged timeframe to better understand the relationships between spatial and temporal factors.

Another challenge in juvenile studies is the accurate identification of lobster life stages. Because crustaceans grow only by shedding their hard exoskeletons during molting, they lose calcified structures that could otherwise be used to determine age. As a result, indirect methods such as length-frequency analysis are commonly applied in life history studies^29^. Apart from the carapace length (CL), other considerations have been proposed to define the juvenile stage, such as activity patterns, foraging behaviors, shelter usage, and ecological roles^30^. Given the data availability in general population surveys, the lobster size distribution based on CL measurements is often used to reflect the life stage. In this study, we also utilized CL measurements as indicators of lobster life stages. Significant annual and seasonal changes in lobster size distribution and frequency have been reported in lobster populations from LFAs 33 and 34, two of the most productive coastal fishing areas in southwestern Nova Scotia. There are concerns that the size at maturity may have shifted to be larger than the legal harvest size (82.5 mm CL)^31^. This highlights the importance of exploring possible reproductive effects on juvenile lobster populations in this region, as such effects may be reflected in the patterns observed in our current study.

Lack of comprehensive information about the long-term trends of the juvenile population and factors associated with it creates a substantial knowledge gap in understanding lobster population dynamics, including in Atlantic Canada. Most importantly, understanding these trends over time is crucial to assessing the juvenile benthic situation and informing effective management decisions. Although LFA boundaries are administrative rather than ecological, they provide a consistent spatial framework for long-term monitoring. This study aimed to examine the long-term trends of juvenile lobster populations, as reflected by the lobster size distribution, in nine Lobster Fishing Areas (LFAs) of Nova Scotia over a period of 20 seasons from 2003 to 2023, and to determine and take into account important predictors.

## Methods

### Study area

**Fig. 1:**
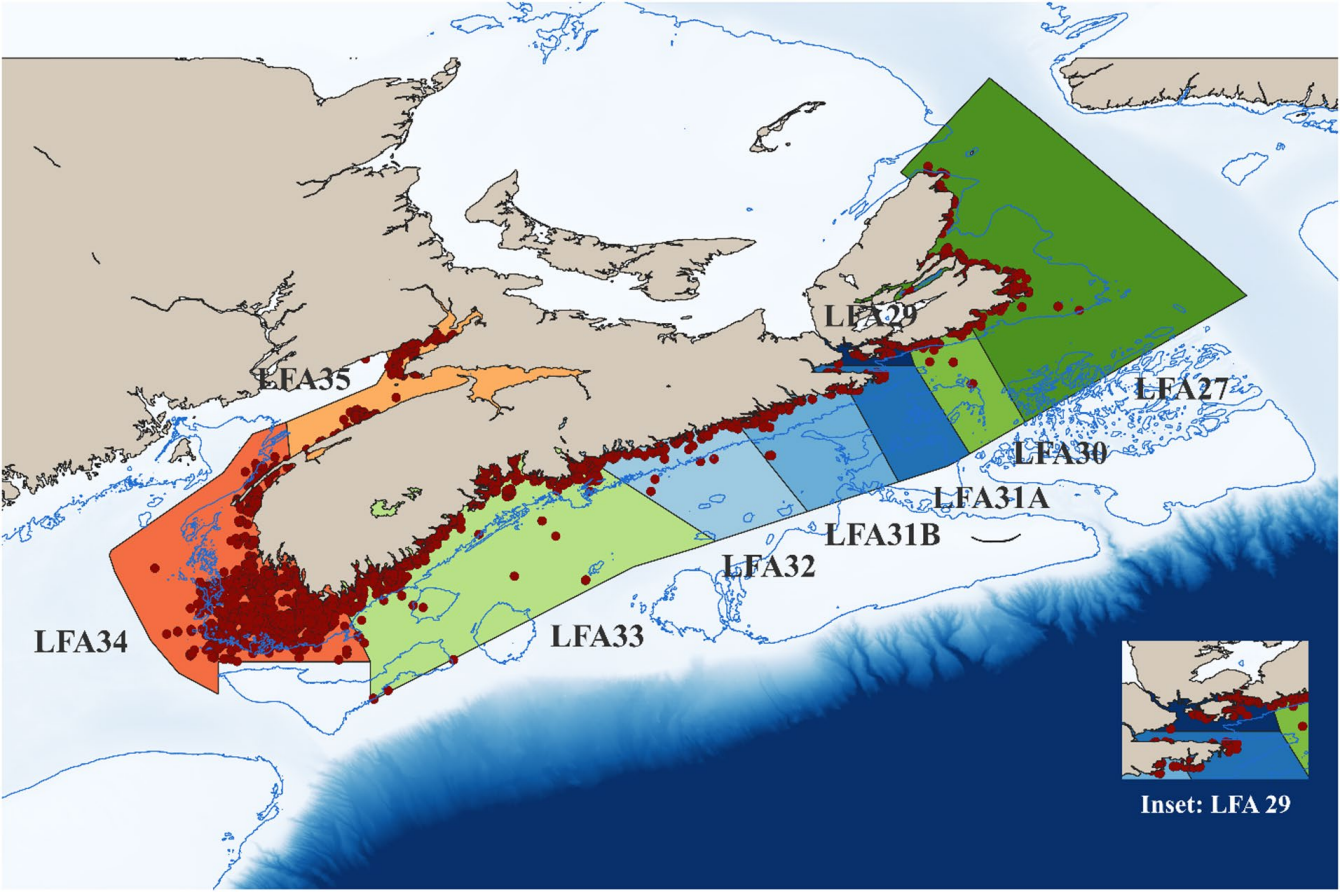
Map of Nova Scotia showing the nine LFAs included in the study. Sampling locations are indicated by red circles. A 100 m depth contour line (blue) is overlaid. An inset map provides a zoomed-in view of LFA 29, a narrow nearshore area that is difficult to visualize in the full map.

Nine LFAs along the Nova Scotian coast of Canada were included in this study, including LFAs 27, 29, 30, 31A, 31B, 32, 33, 34, and 35. A map showing the nine LFAs with the sampling locations is provided (Fig. 1). The administrative boundaries of each LFA were defined within the Atlantic Fishery Regulations, 1985 (Schedule XIII). Nova Scotia’s lobster fishery is under the regulatory control of Fisheries and Oceans Canada (DFO), which enforces tailored regulations through the division of the coast into LFAs. LFA 27 is located north of LFA 30 around northeastern Cape Breton. LFAs 29, 31A, 31B, and 32 are geographically clustered along the eastern shore of Guysborough County. They are characterized by community-based, small-boat inshore fisheries—locally operated and closely connected to coastal communities, with diverse fishing activities conducted in nearshore waters^32^. LFA 33 stretches along the south shore of Nova Scotia, LFA 34 covers the southwestern coastline from Digby Neck to Barrington Bay, and LFA 35 is located in the Bay of Fundy, bordering Nova Scotia and New Brunswick.

Fishing effort in each LFA is regulated through a combination of limited-entry licensing, trap limits, minimum legal sizes, and seasonal closures^33^. As of December 31, 2021, the number and type of licenses issued in each LFA, along with trap limits and minimum legal sizes, are provided in Table S1, adapted from publicly available information on the DFO website^34^. Licensing policies are designed to control exploitation rates and ensure sustainable harvests within each LFA. Trap limits are intended to prevent overfishing and ensure equitable access to the resource. Legal size limits, meanwhile, are set to allow lobsters to reach maturity and reproduce before harvest. Several LFAs have implemented additional measures to align management with local stock conditions, such as maximum hoop size regulations and v-notching—a practice in which egg-bearing female lobsters are marked with a small notch on a tail flap to prevent their removal in future catches.

Lobster fishing seasons are scheduled to avoid disruptions during egg-laying, molting, and hatching periods, while also promoting a more consistent annual supply of lobsters^33,35^. LFA 35 operates during two distinct periods each year: fall (October to January) and spring (April to July). In contrast, lobster fishing in LFAs 33 and 34 takes place from November through June of the following year. For the other LFAs (27, 29, 30, 31A, 31B, and 32), the fishing season runs from April to July. While general seasonal patterns are consistent, specific opening and closing dates may vary slightly between years due to factors such as ice conditions or inclement weather that can delay safe access to the fishing grounds. The exact season dates for each LFA included in this study are provided in Supplementary Table S2.

### Data description

Datasets from the Lobster Recruitment Project 2003–2023 were obtained from the Fishermen and Scientists Research Society (FSRS)^36^. This project involves over 150 volunteer fishers participating in sampling lobsters from nine LFAs in Nova Scotia during lobster fishing seasons. The research traps were placed at fixed locations (2–5 traps per location), with the coordinates for each trap recorded. Participants were instructed to leave their traps at fixed locations throughout the fishing season, returning periodically to check traps and collect data from captured lobsters. All undersized and egg-bearing lobsters caught in the science traps are returned alive to the water, in accordance with the standard handling practices in the fishery. Only legal-sized lobsters are permitted to be retained under the conditions of the science license. As the study design did not explicitly account for the repeated capture of individuals, the mandatory return of undersized and egg-bearing lobsters to the sea likely resulted in some individuals being sampled more than once within the study area.

Traps were typically emptied daily, with a median soak time of one day. However, the interval between checks varied, ranging from as short as half a day to as long as 90 days. Each year, the traps were returned to the same spots to ensure consistency in the data. The traps used in this study were 101.6 × 35.6 × 53.3 cm with 2.5 cm mesh wire without escape mechanisms. The bottom water temperature was recorded using a data logger attached to one of the traps at a specific location. The fishers recorded the size of the lobsters captured using a special measuring gauge, which divided lobsters into 15 size groupings (10 mm CL interval) during 2003–2019 and into 27 size groupings (5 mm CL interval) from 2020 onwards.

This study is restricted to a depth of less than or equal to 60 m where the lobsters are generally found to avoid the influence of extreme depths, which comprises only a small proportion of data (< 1%). The study years were recategorized according to the designated fishing seasons for each LFA to define fishing years that better reflect the sampling periods associated with each season. The year 2020 was excluded due to the inconsistency of the data collected during the COVID-19 pandemic. Additionally, as the trap was wired with 2.5 cm mesh (25.4 mm), lobsters smaller than the mesh width could escape from the trap, biasing against their capture by the sampling method. All lobsters 30 mm CL and smaller, totaling 6,406 individuals (0.40%), were excluded from the analysis to focus on sizes that could be reliably predicted. The recorded bait types used in lobster traps were highly diverse, resulting in a large number of distinct categories, each with relatively few observations. To address this sparsity and facilitate analysis, bait types were consolidated into four broader groups: herring, mackerel, redfish, and others.

### Descriptive analysis

A descriptive statistical analysis was performed to understand the overall characteristics of the data using Stata v.18 (StataCorp, College Station, TX). Heat maps were created using RStudio v.4.4.1 to depict the number of observations at the sampling event level.

### Model outcome variables and the main exposure of interest

The mean CL for each size bin was computed by averaging the lower and upper bounds, i.e., $\:\frac{(lower\:bound+upper\:bound)}{2}\:\:$to generate a variable representing the quantitative outcome of interest. The distribution of mean CL was visualized using a histogram and normal quantile plot.

For the binary outcome, we define “juvenile” lobsters as individuals with a CL below or equal to 60 mm, following the threshold used in a previous study^37^. While this threshold is not intended as a precise maturity marker, it provides a consistent framework for examining trends in smaller size classes. We acknowledge that size at maturity may vary over time and among LFAs; however, applying a fixed threshold offers a practical and consistent basis for comparison across regions and years, given the limitations of available data. A line plot of the proportion of juvenile lobsters was used to describe the temporal pattern over the study years. The combination of LFA and year was taken as the main exposure of interest to explore trends of lobster size and juvenile probability over 20 years of study for each LFA.

### Model building

A directed acyclic graph was drawn using DAGitty v.3.1^38^ to illustrate the potential relationships among multiple factors that contribute to the lobster size, as shown in the supplementary materials (Fig. S1).

Mixed effect linear regression was used to model the quantitative outcome (mean CL), while a mixed effect logistic regression model was applied to analyze the binary outcome of juvenile versus adult status. Both models were fitted using MLwiN v.3.05.

### Determination of hierarchical structure

To reflect the clustering introduced by the sampling design, we created segments to represent lobsters sampled in close proximity in time, space, and environmental conditions. Specifically, samples were assigned to the same segment if they were collected with time gaps less than two months, within a two-kilometer distance (based on recorded coordinates), and with less than two meters of depth variation. This grouping captures shared environmental and temporal conditions that may influence lobster size and the probability of a lobster being classified as juvenile. Each segment represents a distinct time series for a particular location, allowing us to capture local temporal trends in the data. Additionally, the segment structure accounts for discontinuities in the time series that may arise from breaks in sampling, such as the end of the fishing season or the absence of vessel activity in a specific location during certain periods.

**Fig. 2:**
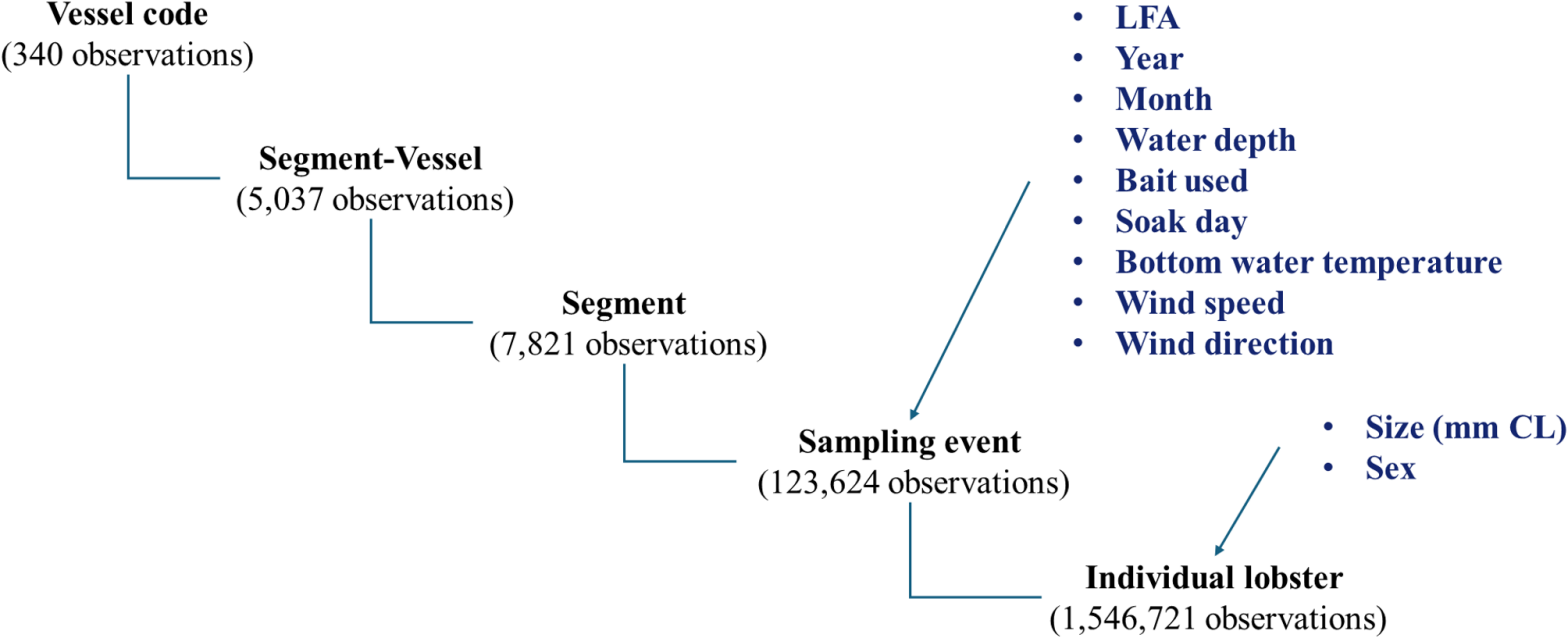
Data structure and variables at each level.

The data follow a five-level hierarchical structure (Fig. 2). Recorded variables and total numbers of observations after removing missing values for lobster size and sex (2.26%) are provided at each level. The highest level is the vessel involved in lobster sampling. To account for (less common) cases where multiple vessels sampled lobsters at the same location and time, a combined Segment-Vessel identifier was created. The segment level captures locations monitored repeatedly by the same vessel within a fishing season. The sampling event represents the clustering of lobsters sampled from the same location by the same vessel on the same day. The number of units at each level and descriptive statistics for the replication at the level above are provided in the supplementary Table S3.

To account for non-independence of outcomes due to this hierarchical structure, four random effects were included in the model: Vessel, Segment-Vessel, Segment, and Sampling event. This approach accounts for similarities among lobsters arising from shared spatial, temporal conditions, and the sampling design.

### Determination of fixed effects

The maximum model included all recorded variables: year, month, LFA, water depth, bottom water temperature, soak day (i.e., the number of days traps were immersed underwater before retrieval), bait type, wind direction, wind speed, and sex. A backward stepwise selection strategy was performed manually. Due to the large dataset (*n* = 1,546,721), variable selection by significance criteria is a less informative approach. The finalized variable selection criteria included: (i) the primary interaction of interest (LFA-Year interaction) and its components; (ii) variables that account for a considerable portion of the sum of squares for the quantitative outcome in simplified analysis of variance (ANOVA) models; and (iii) interesting predictors and interactions supported by relevant literature.

Bottom temperature, wind direction, wind speed, and bait type explained minimal proportions of variance, and were hence excluded from the models. Beyond their limited variance explained, their exclusion is further supported by their positions in the causal diagram—for example, bottom temperature, wind speed, and wind direction occupy positions between LFA-Year interaction and the outcome, acting as intervening variables that do not warrant their inclusion in the analysis. Bait type is related only to the outcome with an unlikely direct association to the LFA-Year interaction. In addition, descriptive screening highlighted a high proportion of missing data for wind variables (17.51% for wind speed and 39.18% for wind direction), which would have substantially reduced the available sample under a complete-case approach. To retain variables that are relatively complete and informative, we excluded these from the model.

Two-way interactions between spatial-temporal factors were modelled, including the LFA-Year interaction of primary interest and the LFA-Month interaction, which is practically relevant due to the variation in fishing seasons (sampling months) across LFAs. Additionally, two-way interactions between water depth and the remaining predictors, including year, month, and LFA were further explored. The strongest interaction was observed between water depth and LFA. Furthermore, due to the well-established association between water depth and lobster sex^31^, the aggregated dataset was stratified by lobster sex to model this three-way interaction. This subset analysis revealed that the depth slopes for males and females differed across LFAs. Therefore, the LFA-Depth-Sex interaction was included in the final model.

Following our variable selection strategy and exploratory analysis described above, three interaction terms (LFA-Year, LFA-Month and LFA-Depth-Sex) and their components were included in the final mixed effect linear regression model. The day of segment, defined as the number of days since fishing began for each segment, was included as a fixed effect and a random slope at the segment level to account for autocorrelation within segments. An additional mixed effect logistic regression model was also fitted using the same terms to better illustrate the probability of sampling juvenile lobsters. Due to convergence issues, two observations of lobsters sampled from LFA 30 in July were excluded from the logistic model.

### Evaluation of model assumptions and residual analysis

Model assumptions were assessed by residual analysis using MLwiN v.3.05. The normality of diagnostic residuals at the lobster level was evaluated using a histogram and normal quantile plot. The plot of diagnostic residuals against fitted values was used to assess the homoscedasticity at the lobster level. For the higher levels, the best linear unbiased predictions (BLUPs) of random effects were checked for normality, and the homoscedasticity assessment of the random effect was carried out by plotting BLUPs of random effects against the fitted values. The model checking for the mixed effect logistic regression was based on the random effects using similar procedures. Extreme observations identified by large diagnostic residuals were inspected to understand why they were poorly fit by the models. As part of the exploratory analysis, model assumptions were evaluated, and random effects at higher levels showed concerning patterns, including deviations from normality and indications of unequal variance. To provide a more cautious analysis and address potential violations of these assumptions, robust standard errors (SEs) were used to improve robustness against non-normality and heteroscedasticity, which may offer substantial improvement, particularly for the SEs of variance parameters^39^.

## Results

### Description of the study population

**Fig. 3:**
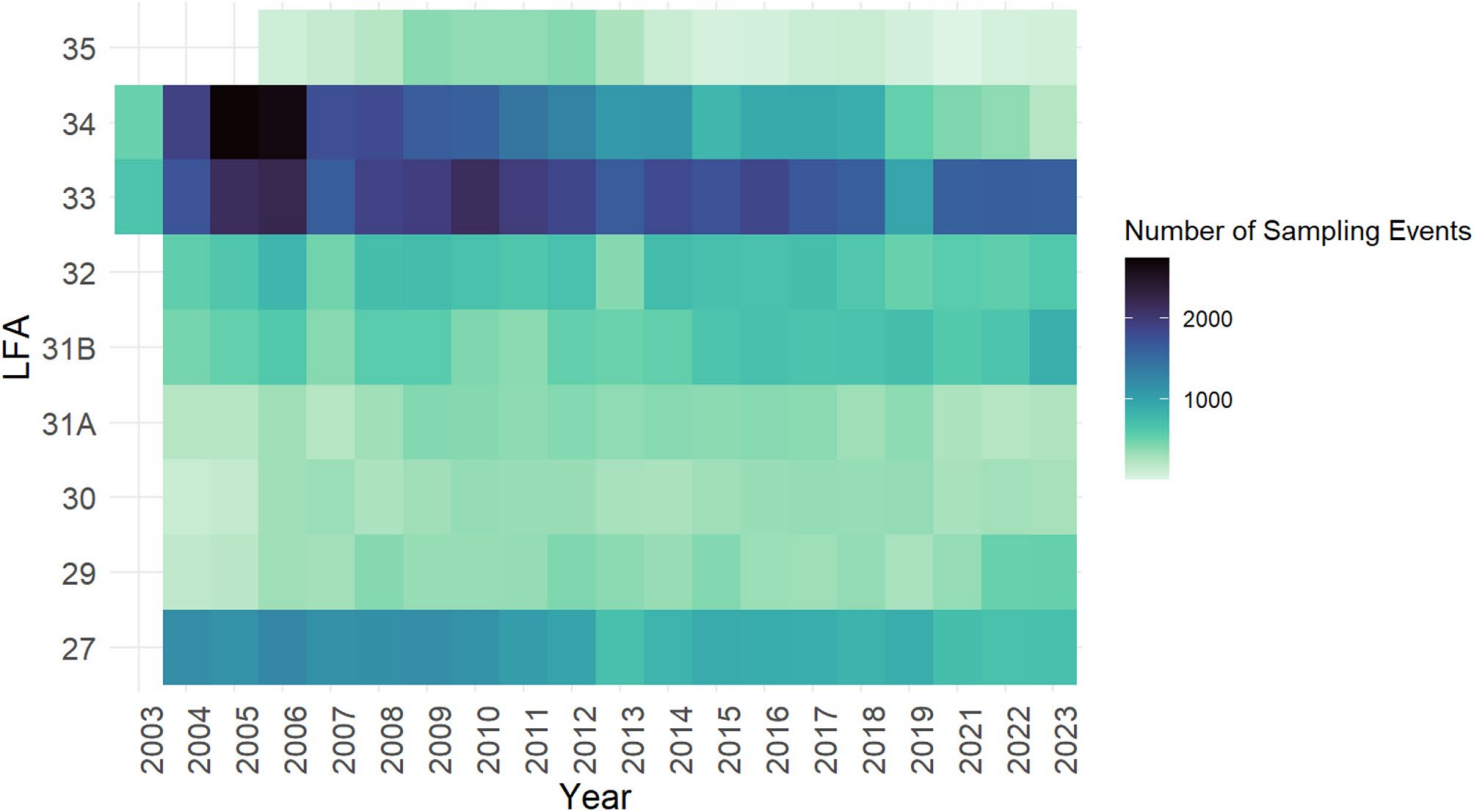
Heat maps summarizing the number of observations at the sampling event level in each LFA from 2003 to 2023.

**Fig. 4:**
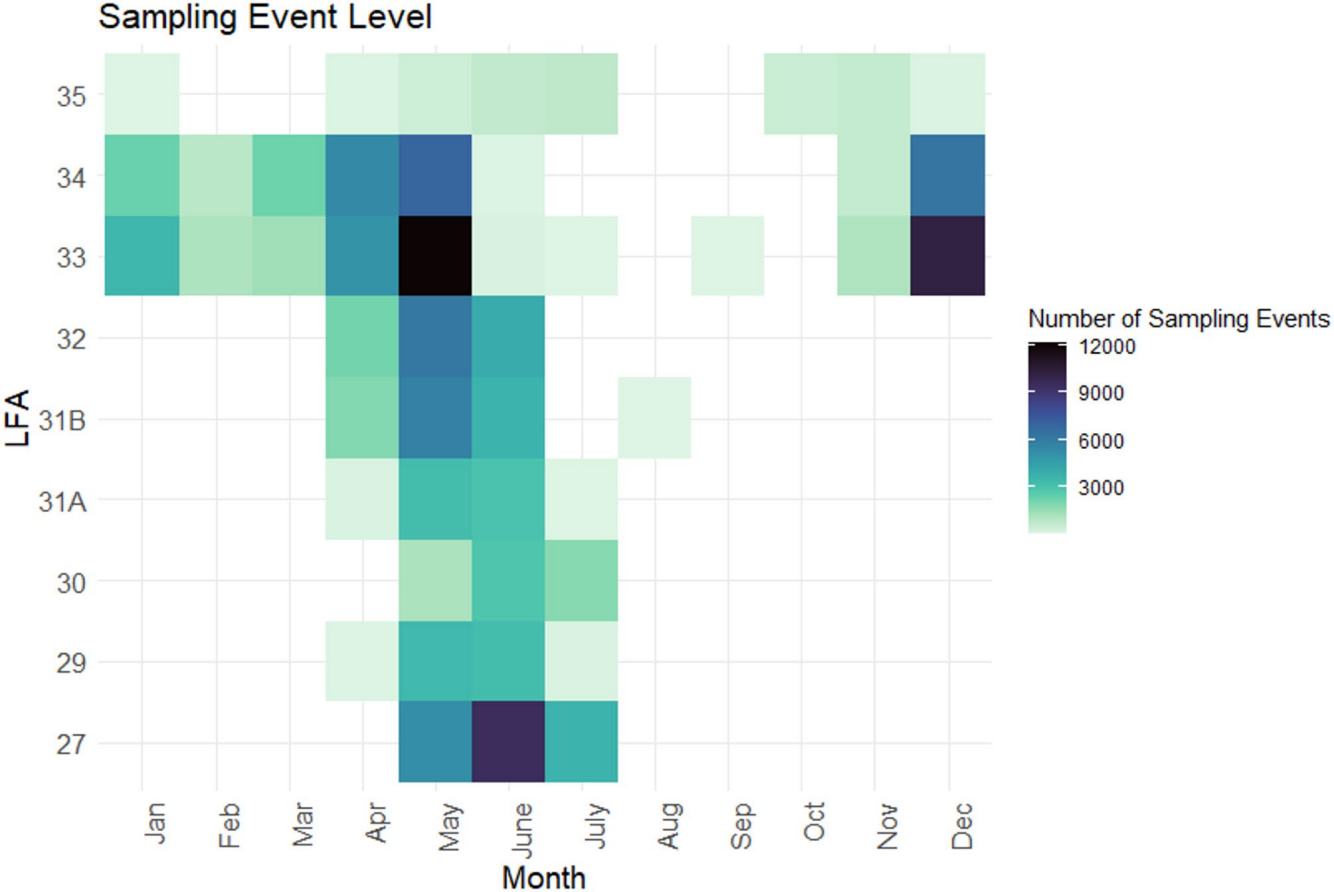
Heat maps summarizing sampling months corresponding to the fishing season for each LFA.

A heat map illustrating the number of observations for each LFA from 2003 to 2023 (Fig. 3) revealed that LFAs 33 and 34, the two largest LFAs in Nova Scotia, accounted for a considerable number of sampling events throughout the study period, although sampling events in LFA 34 declined toward the end of the study. Additional heatmaps for sampling months corresponding to the fishing season for each LFA are presented in Fig. 4.

**Fig. 5:**
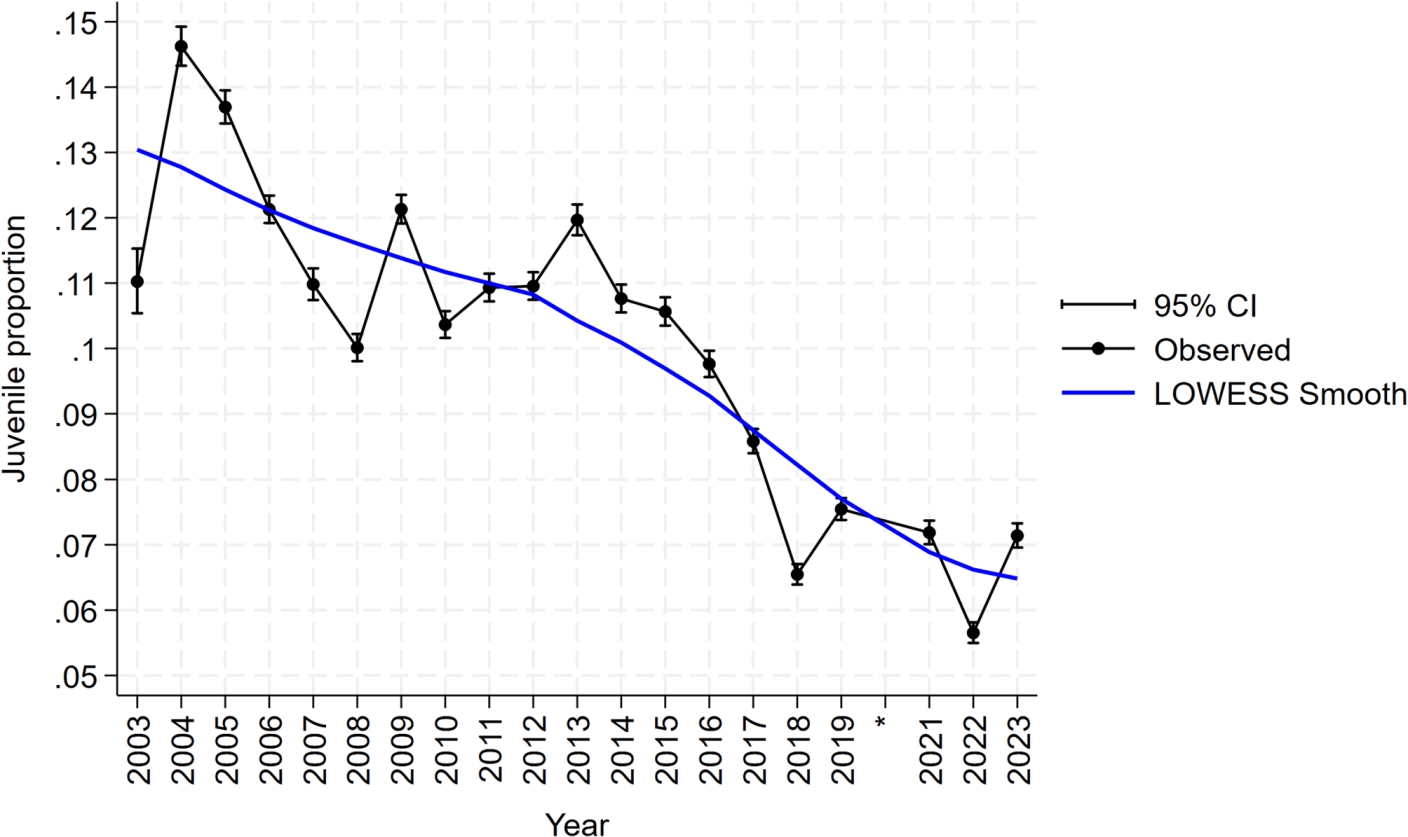
A descriptive line plot presenting the overall proportion of lobsters in the juvenile size class (31–60 mm CL) relative to the total lobsters sampled across nine LFAs from 2003 to 2023, with 95% confidence intervals. A LOWESS smoothing curve is included to represent the overall trend over time.

**Fig. 6:**
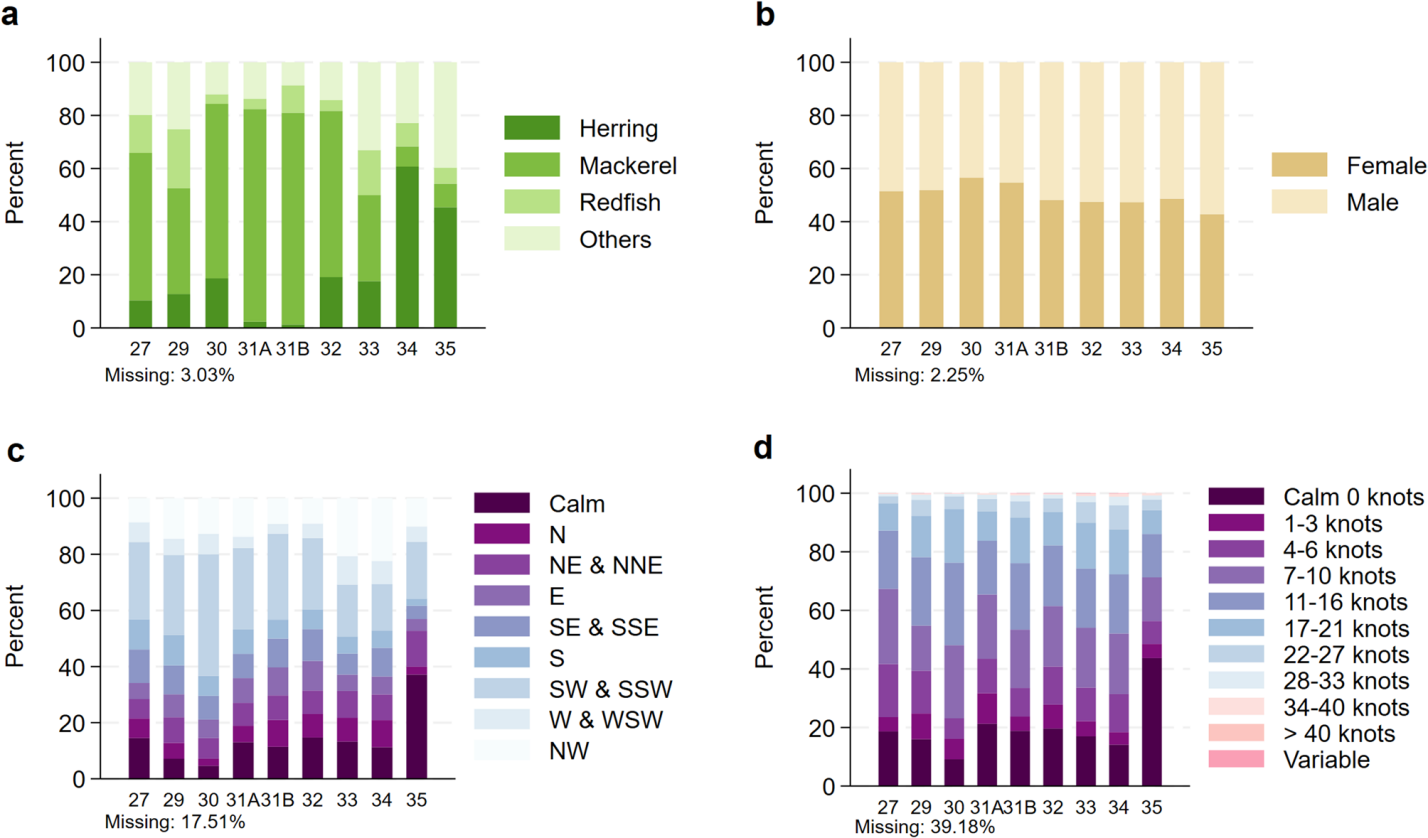
Descriptive summary of categorical variables. Bar charts for bait type, wind direction, and wind speed were created at the sampling event level, while the chart for lobster sex was created at the lobster level. The percentage of missing observations is provided at the bottom of each graph. (**a**) Bait type; (**b**) Lobster sex; (**c**) Wind direction; and (**d**) Wind speed.

A table of descriptive statistics for quantitative variables, including mean CL, bottom temperature, water depth, and soak day is presented in Table 1, with soak day illustrated discretely in the supplementary material (Fig. S3). Overall, juveniles constitute 9.94% of the entire dataset (153,697/1,546,721). The distribution of the quantitative outcome was roughly normal and discrete, with only mean values available for each size bin (Fig. S2). The descriptive line plot of juvenile proportions shows a declining trend over the study years from the highest percentage of 14.62% in 2004 (7,843/53,633) to 7.14% in 2023 (5,328/74,617), the most recent year of study (Fig. 5). Additional plots for each LFA are available in the supplementary materials (Fig. S4). Despite the small number of juveniles captured, which may obscure trends, downward trends were observed in LFAs 27 and 30. In contrast, the other LFAs exhibited fluctuating patterns of juvenile proportions. Distributions of other categorical variables (bait type, lobster sex, wind speed, wind direction) for each LFA are provided (Fig. 6).

**Table 1:** Summary of descriptive statistics for quantitative variables. The descriptive statistics of mean CL were calculated at the lobster level, while the others were calculated at the sampling event level.

Variable	*N*	Mean	Min	Q1	Median	Q3	Max	Missing
Mean CL	1,546,721	77.490	33.000	73.000	78.000	85.500	135.500	35,580
Bottom temperature	127,286	5.582	-8.020	3.680	5.250	7.180	18.000	9,887
Water depth	127,286	10.229	1.000	6.000	8.000	12.000	60.000	0
Soak day	127,286	1.886	0.500	1.000	1.000	2.000	90.000	0

### Mixed effect linear and logistic regression models

**Fig. 7:**
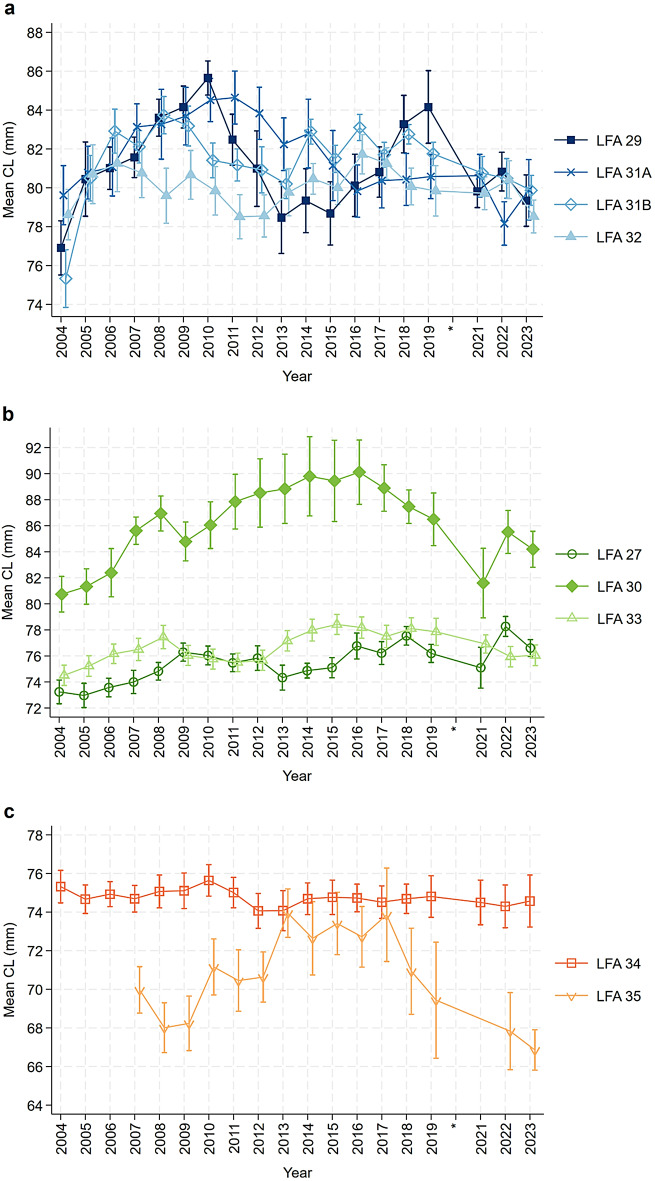
Estimated mean CL (with robust SE) of *Homarus americanus* sampled from different LFAs over 20 years after adjusting for the sampling month and water depth, based on the mixed effect linear regression model. Predictions are fixed to May for all LFAs. (**a**) Water depth fixed at 6 m for LFAs 29, 31A, 31B, and 32; (**b**) Water depth fixed at 8 m for LFAs 27, 30, and 33; (**c**) Water depth fixed at 12 m for LFAs 34 and 35. Data from the entire year of 2020 (indicated with *) were excluded from the analysis due to inconsistencies in data collection during the COVID-19 pandemic.

The main finding of the final five-level mixed effect linear regression model is the significant interaction between year and LFA, illustrated by plots showing estimated trends in mean CL over 20 years across different LFAs (Fig. 7). The distribution of water depth from which males and females were sampled in each LFA was visualized using box plots (Fig. S5). These plots were used to identify the median sampling depth for each LFA, which guided the selection of representative depth values for model predictions while accounting for variability across areas. Based on these depth distributions, three depth scenarios were selected for model predictions: 6 m for LFAs 29, 31A, 31B, and 32; 8 m for LFAs 27, 30, and 33; and 12 m for LFAs 34 and 35. Due to the natural gaps in sampling months throughout the year—where no observations were available outside the fishing season—the month of May was fixed for all the predictions to ensure valid observations across all LFAs. The trends for the other months were examined and confirmed to show no major differences in the patterns. A descriptive summary of average May bottom water temperature by LFA and year is provided in the supplementary material (Fig. S6).

The modelled spatial-temporal effects on the mean CL were demonstrated for each LFA over a 20-year study period in Fig. 7. Overall, the trends in mean CL over time vary between LFAs, making it challenging to identify common patterns for the LFAs with a water depth of six meters (Fig. 7a). LFAs 27 and 33 (scenario with a water depth of eight meters) show a slight upward trend in mean CL over the study period, although with some fluctuations (Fig. 7b), while the predicted mean CL of lobsters sampled from LFA 30 initially rose from 81 mm in 2004 to 90 mm in 2016, before declining to a low of 82 mm in 2021. The variability is also observed in the prediction scenario at 12 m depth, representing conditions for LFAs 34 and 35. In LFA 35, mean CL declined from 2009 to 2013, showed a partial recovery peaking in 2016, dropped to its lowest point in 2017, and then increased steadily, returning to levels comparable to 2007–2009 in recent years. In contrast, mean CL in LFA 34 remained relatively stable throughout the study period (Fig. 7c).

**Fig. 8:**
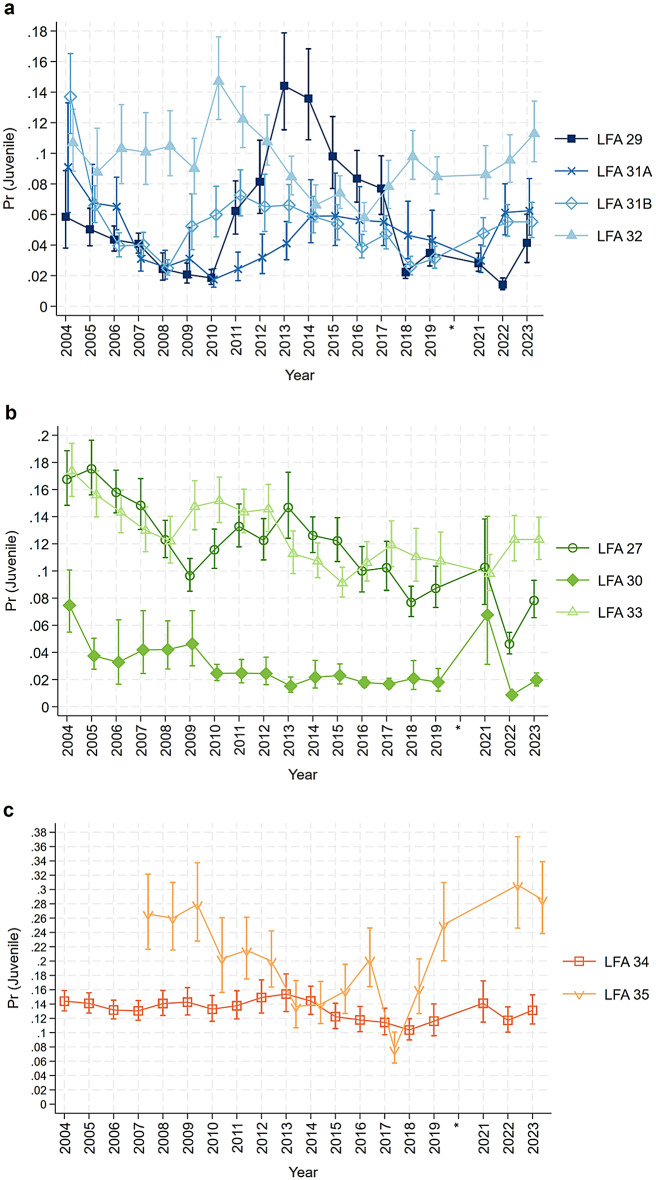
Estimated probabilities of sampling juvenile *Homarus americanus* (31–60 mm CL, representing a smaller size class) from different LFAs over 20 years, after adjusting for the sampling month and water depth, based on the mixed effect logistic regression model. Predictions are fixed to May for all LFAs. (**a**) Water depth fixed at 6 m for LFAs 29, 31A, 31B, and 32; (**b**) Water depth fixed at 8 m for LFAs 27, 30, and 33; (**c**) Water depth fixed at 12 m for LFAs 34 and 35. Data from the entire year of 2020 (indicated with *) were excluded from the analysis due to inconsistencies in data collection during the COVID-19 pandemic.

From the mixed effect logistic regression model, the probability of sampling juveniles is low across all LFAs, with probabilities ranging from just above 0 to 0.3. The overall patterns of juvenile probabilities were inversely related to the estimated mean CL from the linear model. For example, an increase in the mean CL reflected a decrease in the probabilities of sampling juveniles. In LFAs 29, 31A, 31B, and 32, for which a depth of six meters was used in the model predictions, juvenile sampling probabilities exhibit fluctuating patterns throughout the study period (Fig. 8a). In contrast, LFAs 27, 30, and 33—modelled using a depth of eight meters—show a declining trend from the beginning of the study to the most recent year in 2023 (Fig. 8b). Meanwhile, LFA 35 displays a pattern with changes in direction over time, whereas LFA 34 maintains a stable pattern (Fig. 8c). The effects of other important predictors on juvenile probability, including the interaction between LFA and month and the three-way interaction among LFA, depth, and sex, are presented and described in the supplementary materials (Fig. S7-S8). Seasonal patterns (Fig. S7) showed flat to slightly increasing trends in juvenile probability over the course of the fishing season in several LFAs, with LFA 35 exhibiting a declining trend. Depth-related patterns (Fig. S8) varied by LFA and sex; most LFAs showed a decrease in juvenile probability with increasing depth, although exceptions were observed, such as in LFA 30. However, since these interactions are not the primary focus (which is the LFA-Year interaction), they are included here for completeness and reader interest rather than for detailed interpretation.

The estimate with robust SE and proportion of unexplained variance from the mixed effect linear and logistic regression models at day 1 of a segment are summarized in Table 2. Both models suggest minimal clustering at higher hierarchical levels. The lowest-level variance for the logistic model was fixed at 3.29 (π²/3), based on an approximation method using a latent response variable approach^39^. The variance components at the upper levels were small, while a large proportion of unexplained variance was at the lobster level. This demonstrates some similarity in mean CL and juvenile probabilities on a logit scale between individual lobsters within a sampling event. Further interpretations of the random effects parameters are provided in the supplementary materials (Table S4).

**Table 2:** Estimate with robust SE and proportion of unexplained at each level from the mixed effect linear and logistic regression models. For the logistic model, the lowest-level variance was fixed at 3.29 (π²/3) based on the latent response variable approximation.

Random effect parameter	Linear model		Logistic model	
	Estimate (SE)	Proportion of unexplained variance	Estimate (SE)	Proportion of unexplained variance
Vessel level	17.191 (1.977)	0.096	0.672 (0.089)	0.146
Segment-Vessel level	5.963 (0.982)	0.033	0.206 (0.048)	0.045
Segment level	8.913 (0.734)	0.050	0.321 (0.054)	0.070
Day of segment	61.937 (4.892)	-	2.186 (0.375)	-
Covariance	-14.773 (1.638)	-	-0.554 (0.130)	-
Sampling event level	4.444 (0.321)	0.025	0.113 (0.035)	0.025
Lobster level	141.673 (5.157)	0.795	3.29	0.715

## Discussion

This study highlighted 20-season trends in lobster size changes by examining mean CL and modelling the probability of lobsters falling within the juvenile size class (31–60 mm CL). The investigation revealed the dependency of these annual lobster size and probability patterns on the LFAs. The distributions of lobsters sampled from different water depths indicate three common depths specific to groups of LFAs, which are shaded using the same base color in the figures presenting model results.

In LFAs 29, 31A, 31B, and 32, the lobster catch was carried out in the shallowest water with a median depth of six meters. These four LFAs are adjacent, with LFA 29 being the LFA closest to shore, as it is bordered by the shore and surrounded by LFA 30 and 31A. The fluctuating patterns in the mean CL and probability of sampling juvenile lobsters among these four regions suggest the ability to recover from depleted juvenile populations. In the same way, the impacts on the juvenile lobster population do not exhibit distinct trends, possibly due to small-scale fishing activities in local communities not extensively exploiting harvestable lobster resources, nor markedly disturbing the juvenile behaviors and density^40–42^. In 2001, management adjustments were introduced to these areas including increasing the minimum legal size (MLS) by 1.5 mm from 81 to 82.5 mm in LFAs 31B and 32, along with a requirement for each license holder to release 50 kg of large non-ovigerous females back to the fishing ground annually, and specifically in LFA 31A, the MLS was elevated by 5 mm, from 81 mm to 86 mm CL, and non-ovigerous females measuring between 114 mm and 124 mm CL are not permitted to be retained; they must be released if captured^43^. Following the management adjustments in 2001, we observed an initial increase in the mean CL of sampled lobsters across LFAs 29, 31A, 31B, and 32 starting around 2004. These increases, however, were not consistently sustained, and all four LFAs showed varying fluctuations in mean CL over time. This pattern corresponded to a general decrease in the probability of encountering juvenile lobsters. Juvenile probability began to rise again approximately eight years after the management changes, though this was followed by later declines. These variable trends make it difficult to draw clear conclusions about long-term outcomes, highlighting the importance of continued monitoring to help avoid potential declines in juvenile presence, as seen in LFA 29 during the 2004–2010 period.

The second group of LFAs, where lobsters were sampled in relatively deeper waters (with model predictions fixed at 8 m), shows increasing trends in mean CL across all three LFAs: 27, 30, and 33. Within LFA 27, while a relatively small subset of license holders has significant landed value from other species, most are highly dependent on lobster^34^. The juvenile lobster situation in LFA 27 reported in our study shows a declining proportion of juveniles. This pattern is similar to LFA 33 up until the last two years, after which the trends diverge. Focusing on LFA 27, this finding aligns with previous research indicating that the size at which half of the females reach maturity (CL₅₀) has decreased by approximately 30 mm over several decades (1935–2007), with a significant decline reported^44^. Although females maturing at smaller sizes can increase overall egg production, the quality of these eggs is generally poorer. Eggs from younger, smaller females are typically smaller, have lower nutritional reserves, and experience delayed hatching compared to those from larger females, which negatively impacts egg viability and juvenile recruitment^45^. This decline could be driven by rising ocean temperatures and high fishing pressures from commercial fishing, which exert selective pressure on females to reproduce earlier before entering intense fishery^46^. In LFA 30, egg production per recruit was estimated at around 2%–7% of the unfished population, which remains below the Fisheries Resource Conservation Council (FRCC) target of 5%^47^. This issue persists in LFA 30, as reflected by generally low juvenile proportions over time. Notably, LFA 30 exhibited distinctive trends in mean CL, rising from 80.5 mm in 2004 to 90 mm in 2016, followed by a decline over the subsequent decade. Juvenile probability also declined from 0.08 to a low of 0.02 in 2013. Although there were periodic peaks, such as in 2021, juvenile probabilities have remained relatively low in recent years, with the most recent value returning to 0.02 in 2023. In 1997–2002, LFA 33 adopted several management measures, including voluntary v-notching, increasing the MLS from 81 mm to 82.5 mm, and releasing lobsters with missing claws in response to the Atlantic-wide management plan aimed at increasing egg production per recruit^48^. Unexpectedly, our study did not detect a positive impact from these measures. Despite a reasonable follow-up period of about eight years or more, during which the progeny of lobsters was expected to benefit from the management plan and reach the minimum legal size^49^, our results did not show a consistent or sustained increase in the probability of encountering juveniles in LFA 33. While it is tempting to consider whether adjacent LFAs along the eastern shore may have experienced indirect benefits through larval drift, the observed patterns are variable and fluctuating, with no clear connectivity-driven effect. Given the complexity of larval dispersal and the limitations of the data, we take a cautious approach in interpreting these trends and acknowledge the uncertainty surrounding potential links between management measures and juvenile dynamics across LFAs. Consistent with the 2021 lobster stock status update, it is suggested that decreased catch rates may be due to reduced survival and limited habitat availability for juveniles^50^. Although the 2022 report indicates that LFA 33 is currently in a healthy zone^5^, exploitation in LFA 33 has not been effectively monitored since the fishery shifted to more offshore areas, increasing the risk of undetected stock exploitation^50^. A previous study on lobster sex ratios reported a decline in the probability of sampling females in LFA 33 between 2010 and 2019, which may indicate a negative impact on future reproductive success^51^. The gradual decrease in juvenile probability observed in recent years, following a peak in the early part of the time series, may reflect, in part, the consequences of reduced female proportions. Although the trend is relatively modest, it underscores the importance of continued monitoring and potential adjustments to management policy for this LFA.

For LFAs 34 and 35, where lobsters were sampled at a median depth of 12 m, the mean CL and juvenile probability of lobsters sampled from LFA 34 have shown remarkable stability over the past 20 years. This stability suggests resilience in the juvenile lobster population and may reflect a combination of effective management measures and favorable environmental or oceanographic conditions. Given the extensive spatial coverage of sampling within LFA 34, including both inshore and offshore areas, some spatial heterogeneity in lobster populations may exist. Further investigation into spatial and environmental factors would help clarify the drivers of this observed stability, despite high lobster landings in the area. This finding is consistent with a previous study that reported the highest relative abundance of pre-recruits in southwestern Nova Scotia from FSRS traps^52^. The overall pattern of increasing commercial biomass and decreasing level of exploitation during the study period from several surveys also supports that the lobster stock in LFA 34 has been in a healthy state^6^. Lobsters in LFA 34 demonstrated robust reproductive potential from 2010 to 2019, as evidenced by an increased probability of encountering females, which remained consistent throughout the study years^51^. Besides, an inshore ecological survey conducted in LFA 34 in 2007 highlighted the depletion of large predatory groundfish, such as Atlantic cod (*Gadus morhua*), alongside increased lobster recruitment. The survey concluded that changes in predator abundance may have contributed to the growing lobster stock, allowing lobsters to expand into new habitat areas across various water depths^53^. Reduced predation risk might have a positive impact on juvenile populations by lowering direct consumption and minimizing non-consumptive effects on behavior, such as movement patterns, shelter use, and foraging^54^.

LFA 35 is the LFA located exclusively in the Bay of Fundy included in this study. Our data indicate a notable sharp rise in mean CL occurred between 2008 and 2013, which synchronized an increase in recruit abundance from the trawl survey during the same period^7^. Juvenile probability in LFA 35 has shown considerable variability over time, with a notable increase since 2018, reaching the highest levels observed across all LFAs. However, between 2008 and 2013, fecundity in the Bay of Fundy declined by 31% overall, with an annual decrease of 8%–10%^55^. This earlier decline coincided with lower juvenile probabilities in LFA 35, raising concerns at the time about long-term population sustainability. Similar to LFAs 29, 31A, 31B, and 32, trends in LFA 35 for mean CL and juvenile proportion lack a consistent pattern, complicating efforts to identify underlying drivers. A previous study reported high variability in lobster size-frequency distributions specifically within the Bay of Fundy, covering LFAs 35 and 36^56^. Geographic variation in lobster size is expected and has been attributed to differing levels of exploitation and variations in benthic productivity between the upper and lower Bay. For example, the rocky and silty substrates in the lower Bay are considered more favorable, supporting successful settlement and survival of juvenile lobsters^57,58^. Although LFA 34, which also encompasses parts of the Bay of Fundy, may also exhibit spatial heterogeneity, comparable published evidence documenting variability in lobster size distribution or juvenile probability is limited. As noted earlier, trends in LFA 34 have remained relatively stable over time.

Based on the variance function from the random slopes model, the series shows reasonably constant variance for the first 50 days; after that point, the variance increases. However, the distribution of segment durations is strongly right-skewed, with a median of 22 days, so most series do not reach the 50-day threshold where this shift occurs. In warm-season LFAs, short-term stability of variance likely reflects juvenile lobster biology, as juvenile molting is periodic and largely synchronized during the summer, with most individuals molting no more than once within a short time frame^59^. Larger lobsters molt only once per year or less and tend to show strong site fidelity over short periods, such as within 30 days in a tag-recapture study^42^, which helps maintain a stable upper end of the size distribution. When segment duration exceeds 50 days, individuals at different growth stages appear in the catch. Juveniles may molt more than once, and larger lobsters may shift locations, as shown in studies where adult lobsters were recaptured farther from their release sites over time^30^. These changes may allow more small juveniles to enter traps, particularly in areas where large lobsters no longer dominate. In cold-season LFAs like LFAs 33 and 34, low temperatures suppress molting and reduce movement, so growth does not seem to account for the increase in variance after 50 days. Instead, behavioral and social factors may explain the pattern. Larger lobsters tend to compete more aggressively for access to confined spaces, using their size and claw strength to dominate encounters. Lobsters above 60 mm CL are more likely to exclude smaller individuals from such spaces, while those below 60 mm CL rarely show aggressive behavior or engage in direct competition^30^. In traps where large legal-sized lobsters have been removed due to accumulating fishing activities over nearly two months, smaller lobsters likely co-occur across a wider range of sizes.

In parallel, we also observe a decrease in mean size over time within segments. This suggests that larger, harvestable lobsters are caught earlier, which leads to a gradual shift toward smaller individuals as the segment advances. A behavioral explanation may also apply: larger lobsters tend to enter traps first to compete for food, while smaller ones often wait, especially when larger individuals are already inside. Although one study reported that pre-recruit lobsters entered traps more quickly than legal-sized individuals, it also showed a substantial increase in the catch of legal lobsters after about 24 h of soak time^9^. Since our sampling used soak times of one day or more, traps in the early days of each segment likely held a higher proportion of larger lobsters.

In general, trap-based surveys are characterized by saturation effects, whereby traps can become filled with lobsters, limiting their ability to capture additional individuals during a soak period. These surveys also exhibit size selectivity, as trap design tends to favor the capture of lobsters within certain size ranges. Research traps without escape vents were used in this study to encourage the capture of lobsters of all sizes, including pre-recruit lobsters. A ventless trap has the potential to catch up to ten times more lobsters than standard traps with the same one-inch wire mesh^60^. However, it was suggested that ventless trap surveys may overestimate the mean CL compared to the more accurate scuba transect surveys^61^, likely because small juvenile lobsters can escape through the trap mesh and are less likely to enter the trap when adult lobsters are present^9,62,63^. Size-selective trap catches may result in underestimation of juvenile populations. To minimize the effect of size selectivity, smaller juveniles (≤ 30 mm CL) were excluded from the analysis. Moreover, using traps can potentially introduce selection bias in sampling juveniles because their shelter-dependent behaviors affect their catchability when small juveniles spend significantly less time foraging and more time within shelters, making them less likely to be captured in our research traps^26,64,65^. Juvenile settlement is also associated with our main exposure (LFA and year variability), which encompasses both temporal and geographical factors. This is because oceanographic conditions, substrate, shelter availability, conspecifics, and predation risk vary significantly across different spatial and temporal contexts^66–71^.

The validity of our study is limited to the lobster population in nine LFAs of Nova Scotia. This limitation arose from the trap-based sampling method and the inherent impracticality of random sampling in studying marine populations. Additionally, the study specifically targeted juvenile lobster populations with participants instructed to fish research traps in areas known to have undersized lobsters. This targeted approach may introduce bias relative to the overall size distribution of the population. Although the source population was restricted, this approach practically promoted sufficient catch of juvenile lobsters, which can be rare in some locations. Given these limitations, we recognize that this study is not well suited for estimating lobster abundance or biomass. Instead, the analyses focus on size distribution, which aligns more closely with the objectives and design of the sampling methods used. However, the present study only included the data during the fishing season. Therefore, we recommend that future research incorporate data collected outside this period to better understand how juvenile lobsters behave in the absence of fishing pressure.

Given the rising concerns about climate change and its impact on lobster fisheries, bottom water temperature has been widely discussed as a key factor influencing the lobster size and juvenile population in previous studies^20,46,72^. However, our study found that bottom water temperature, though a potential predictor, explains only a small proportion of the variance in our models. This is likely because its effect is overshadowed and partly represented by the complex relationships among year, month, LFA, and water depth. Additionally, the bottom temperature measured at a sampling time may not appropriately capture its impact on mean CL or juvenile probability, which may require a longer exposure period to become evident. Therefore, using longitudinal temperature data is recommended to better quantify causal associations.

It’s important to recognize that LFA boundaries are administrative constructs and may not precisely correspond to ecological gradients or habitat discontinuities. Consequently, differences observed among LFAs should be interpreted with consideration of both environmental variability and regulatory frameworks. Previous research off southwestern Nova Scotia—covering LFA 34—indicates that lobsters are highly detectable on smooth substrates (sand, gravel, and cobble) but less visible on rougher, boulder-filled seafloors, where they are often found sheltering^73^. In contrast, habitat studies in eastern Nova Scotia are limited. While habitat-based ecological analyses could provide valuable complementary perspectives, using LFAs as the unit of analysis remains the most practical and relevant approach for management, given that regulatory measures are established and enforced solely at this scale.

The temporal effects on mean CL and juvenile proportions varied between LFAs, but demonstrated some patterns based on the median depth of the studied LFAs. It is also likely that additional unmeasured variables, such as habitat suitability, local fisheries practices, the implementation of management measures, and oceanographic conditions, contribute to the observed patterns. Including these factors in future research may provide a more comprehensive understanding and support more effective, area-specific management strategies.

## Conclusion

Despite some limitations and uncertainties, this study provides a comprehensive overview of long-term trends in mean CL and the assessment of juvenile lobster populations over 20 years. The overall proportion of juvenile lobsters sampled during the study period was relatively low at 9.94%, with variation across LFAs ranging from 3.21% to 13.51%. Annual values across all LFAs declined from 14.62% in 2004 to 7.14% in 2023, with the lowest at 5.65% in 2022. Our findings reveal that the dynamics of juvenile populations are more dependent on specific LFAs than previously documented. As LFAs are designated for management purposes, this study highlights the resilience of juvenile populations in some areas, suggesting that management measures have been effectively implemented. However, notable declines in certain areas point to the need for improved monitoring of performance and compliance with their management strategies.

## Supplementary Information

Below is the link to the electronic supplementary material.

Supplementary Material 1

## Data Availability

The data that support the findings of this study are available from the Fishermen and Scientists Research Society (FSRS) but restrictions apply to the availability of these data, which were used under license for the current study, and so are not publicly available. Data are however available from the authors upon reasonable request and with permission of FSRS.
