# Supplementary material for: Long-term trends in juvenile American lobster populations across nine lobster fishing areas in Nova Scotia, Canada
Source: Sci Rep. 2025 Nov 27;15:42444. doi: 10.1038/s41598-025-26491-2 (PMC12661040; doi:10.1038/s41598-025-26491-2)
Supplement: Supplementary file 1 — Supplementary Material 1 [file 41598_2025_26491_MOESM1_ESM.docx]

**Supplementary materials**

**Table S1** Number and type of lobster licenses by LFA as of December 31, 2021, including trap limits for Category A licenses and minimum legal sizes. License types include Category A (full-time), Category B (part-time), and communal commercial. Category B licenses are permitted up to 30% of the Category A trap limit, while licenses fished under partnership or stacking arrangements are allowed up to 150%. Source: Adapted from publicly available information on the DFO website [34].

| LFA | Category A | Category B | Communal commercial | Total | Trap limit | Minimum Legal size (mm) |
| --- | --- | --- | --- | --- | --- | --- |
| 27 | 489* | 11 | 14 | 514 | 275 | 82.5 |
| 29 | 53 | 4 | 5 | 62 | 250 | 84 |
| 30 | 20 | 0 | 0 | 20 | 250 | 82.5 |
| 31A | 68 | 3 | 0 | 71 | 250 | 82.5 |
| 31B | 70 | 0 | 0 | 70 | 250 | 82.5 |
| 32 | 146 | 4 | 6 | 156 | 250 | 82.5 |
| 33 | 635 | 25 | 20 | 680 | 250 | 82.5 |
| 34 | 943 | 0 | 36 | 979 | 375/400 | 82.5 |
| 35 | 73 | 2 | 19 | 94 | 300 | 82.5 |

* Includes 33 licenses based in the Gulf Region. Source of asterisked information: Maritimes Region Licensing Summary Report (LS4041A) and Gulf Region Licensing Office.

**Table S2** Specific fishing season dates for each LFA included in this study

| LFA | Specific Dates of Fishing Season |
| --- | --- |
| 27 | May 15 – July 15 |
| 29 | April 30 – June 30 |
| 30 | May 19 – July 20 |
| 31A | April 29 – June 30 |
| 31B | April 19 – June 20 |
| 32 | April 19 – June 20 |
| 33 and 34 | Last Monday of November – May 31 (following year) |
| 35 | Oct 14 – Dec 31 (Fall) Last day Feb – July 31 (Spring) |

**Table S3** Number of units at each level and descriptive statistics for the replication at the level above

| Level | Number of units | Replication at level above | |
| --- | --- | --- | --- |
|  |  | Mean | Range |
| Vessel code | 340 | - | - |
| Segment-Vessel | 5,037 | 14.8 | 1-353 |
| Segment | 7,821 | 1.6 | 1-39 |
| Sampling event | 123,624 | 15.8 | 1-157 |
| Lobster | 1,546,721 | 12.5 | 1-251 |


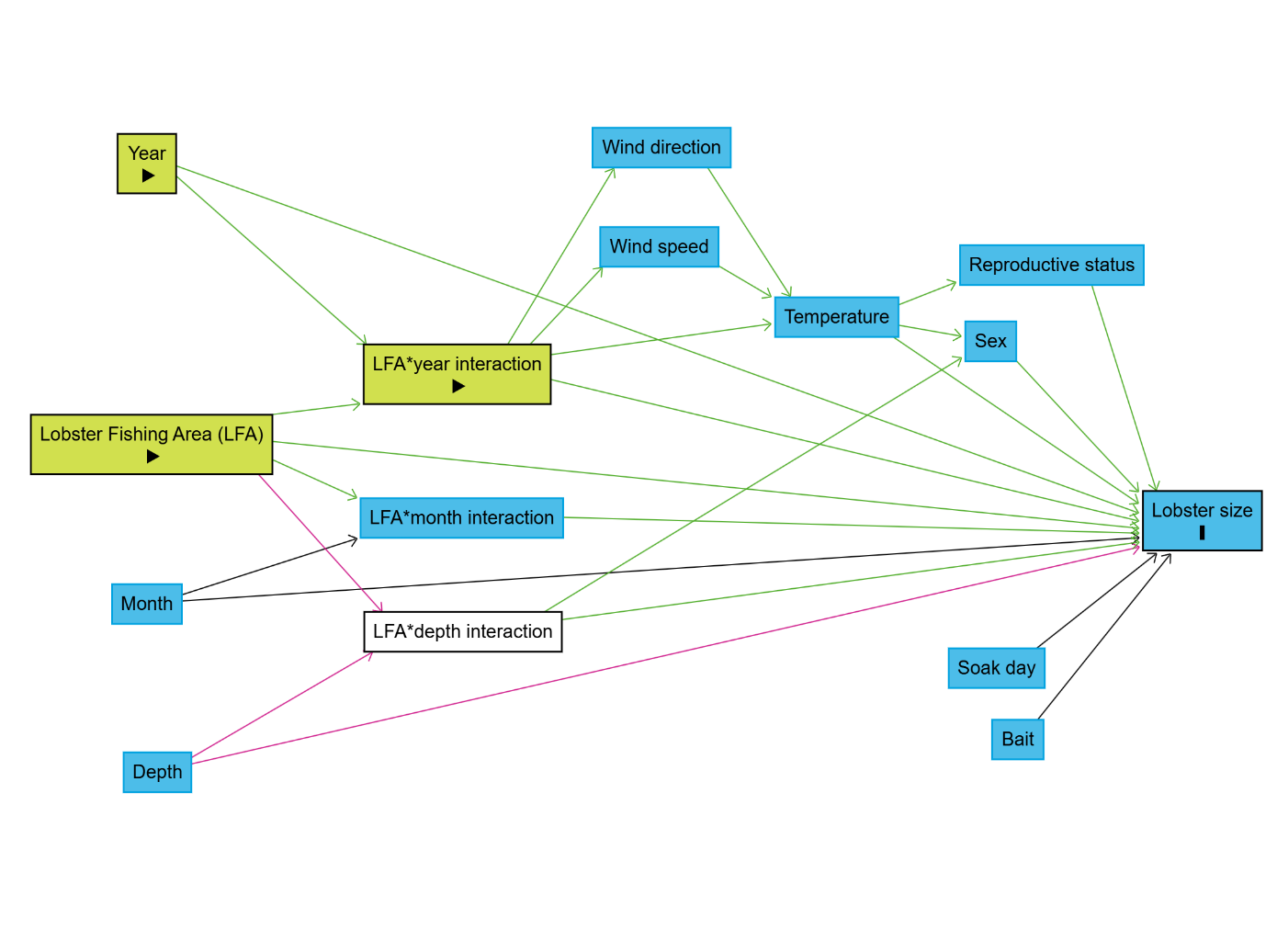


**Figure S1** A directed acyclic graph showing the potential relationships among multiple factors that contribute to the lobster size.


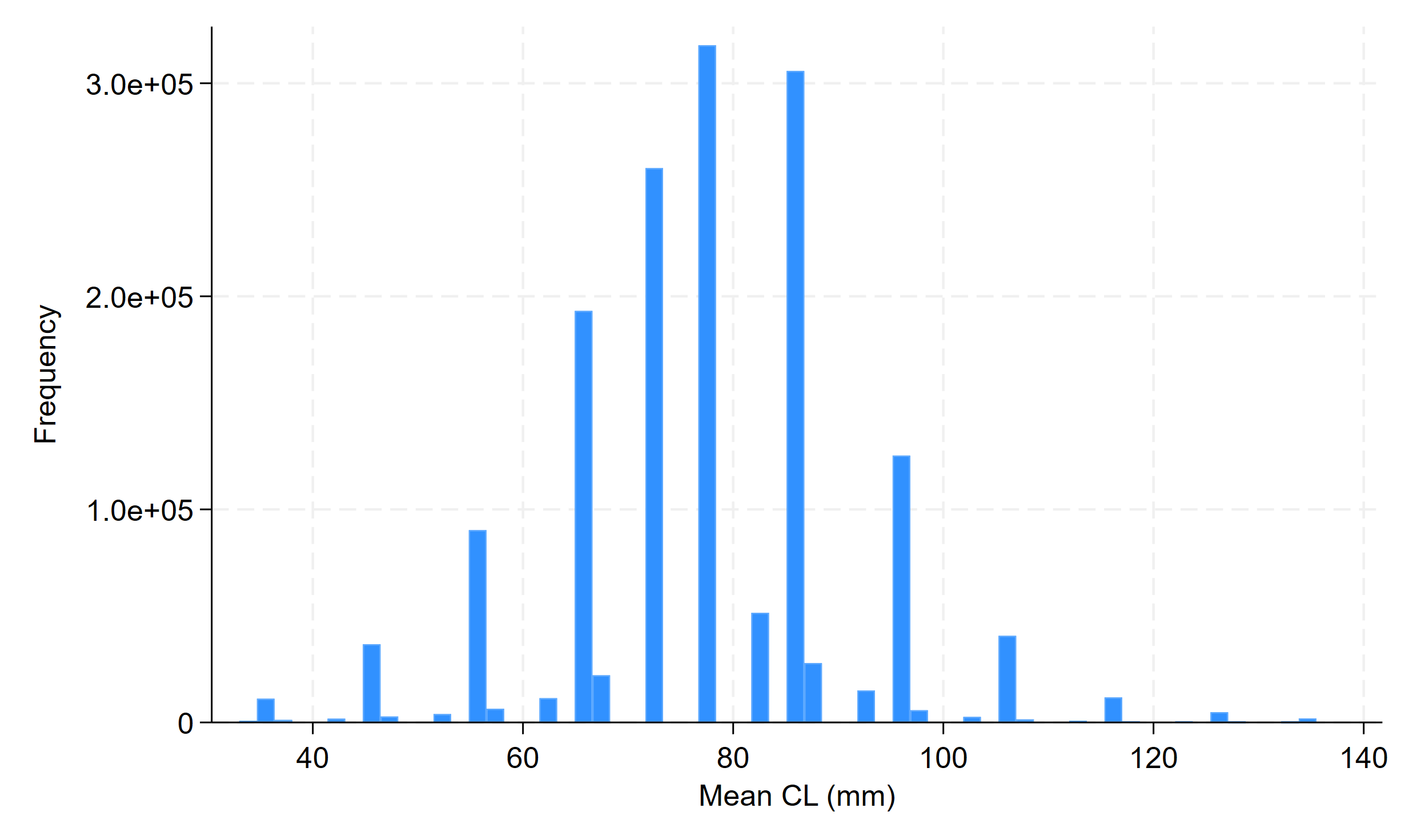


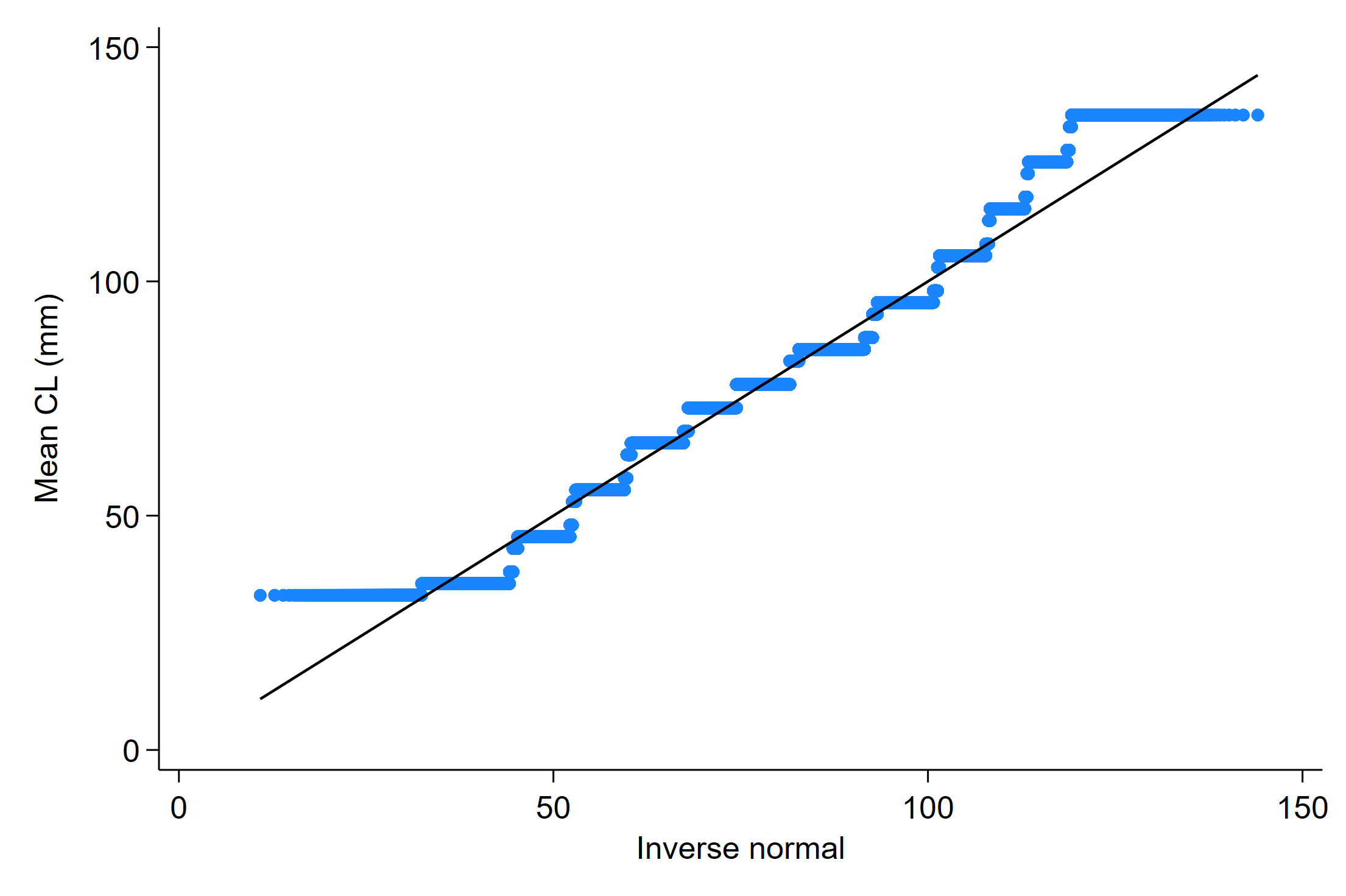


**Figure S2** Histogram and normal quantile plot of mean CL.


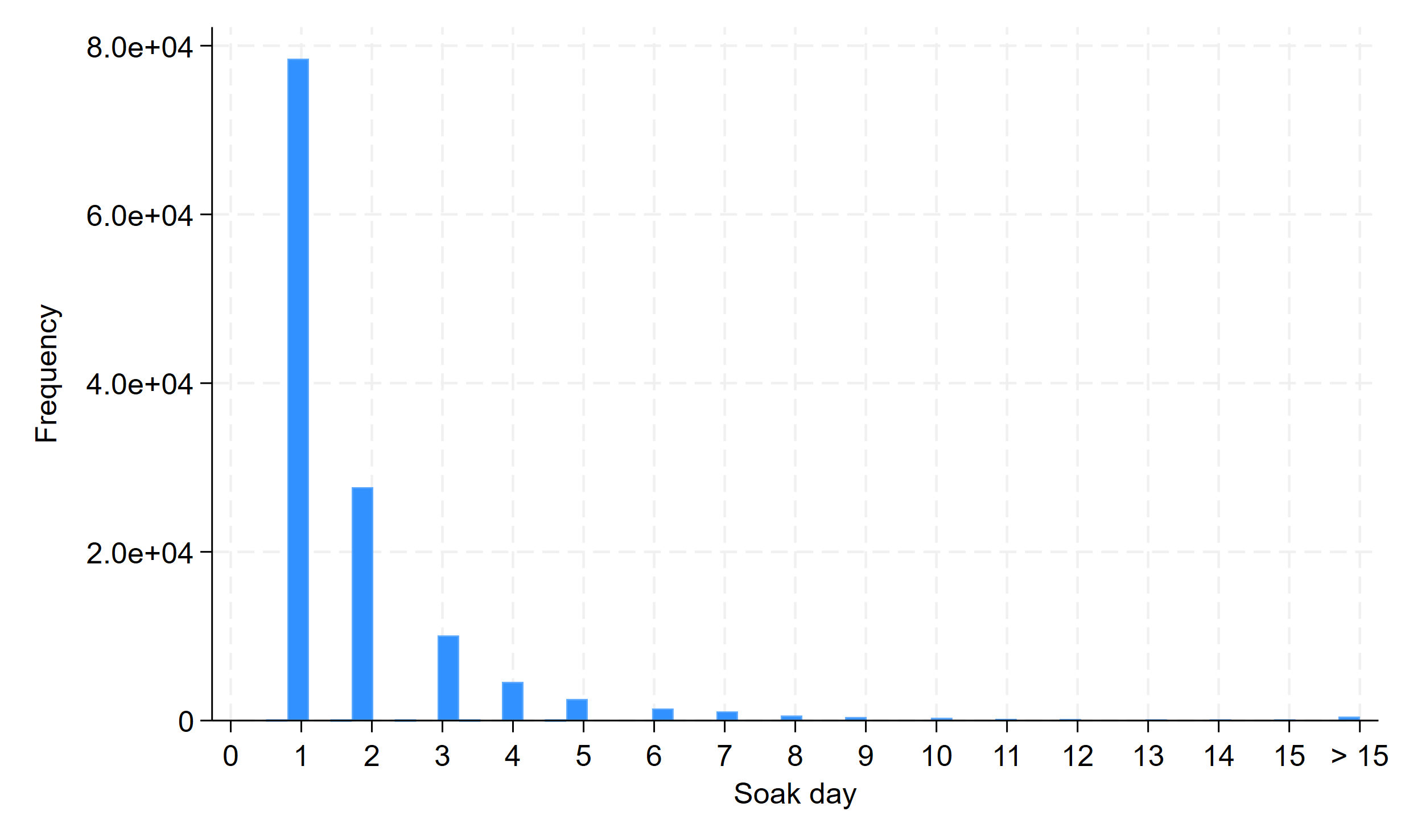


**Figure S3** Histogram of soak day.

**
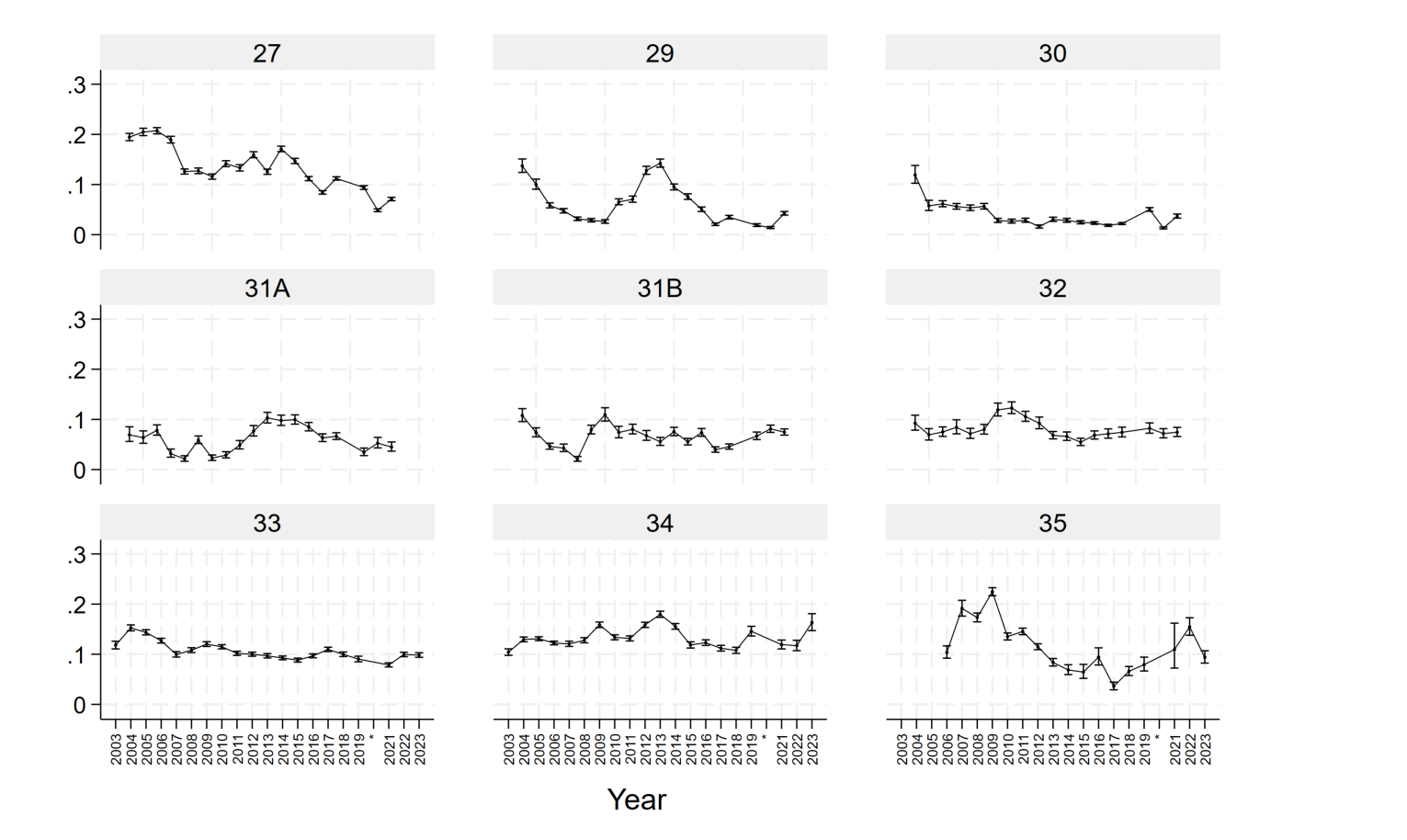
Figure S4** Descriptive line plots presenting the proportion of lobsters in the juvenile size class (31–60 mm CL) with 95% confidence intervals from 2003 to 2023 by LFA.


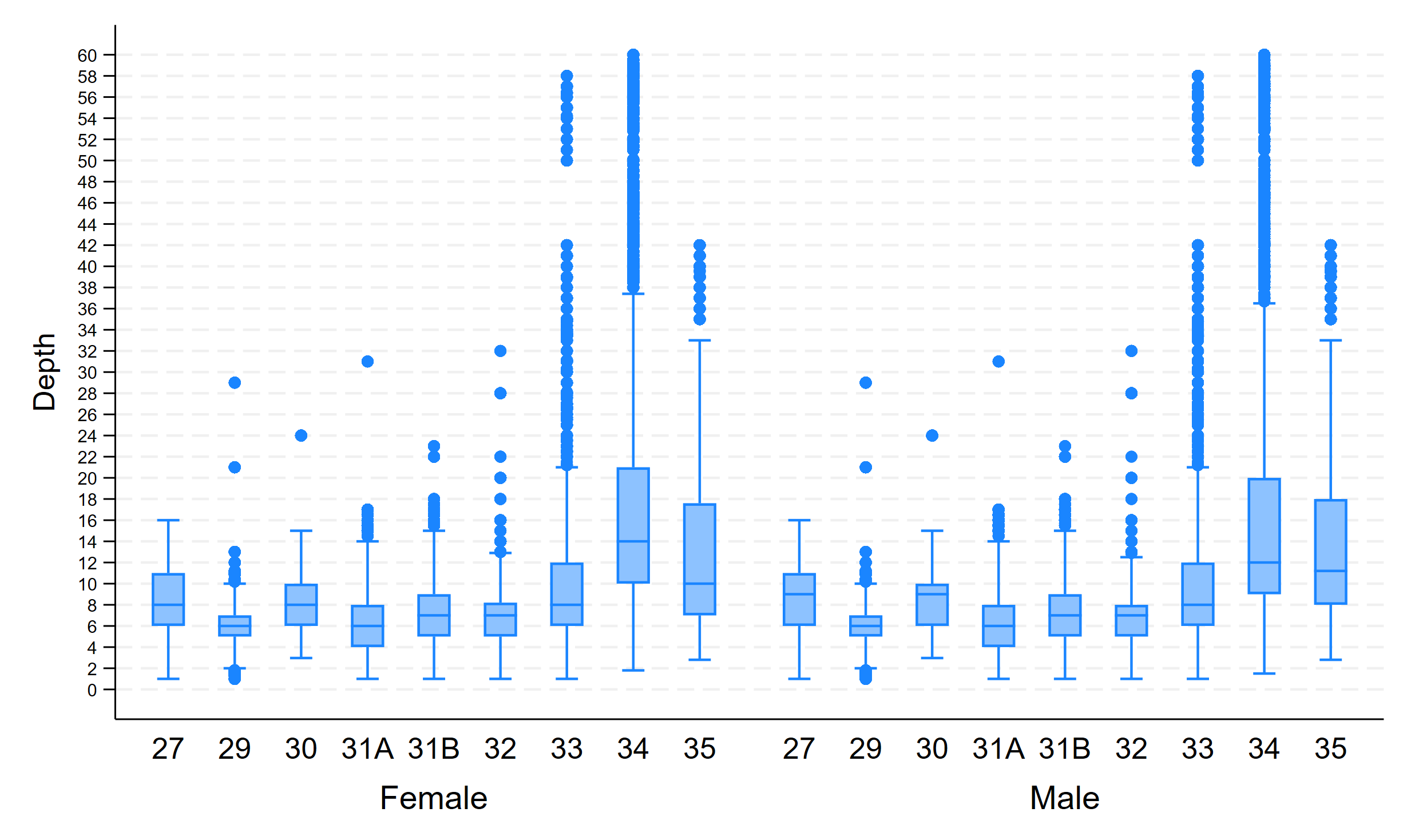


**Figure S5** boxplots of water depth where *Homarus americanus* were sampled for each LFA by sex.


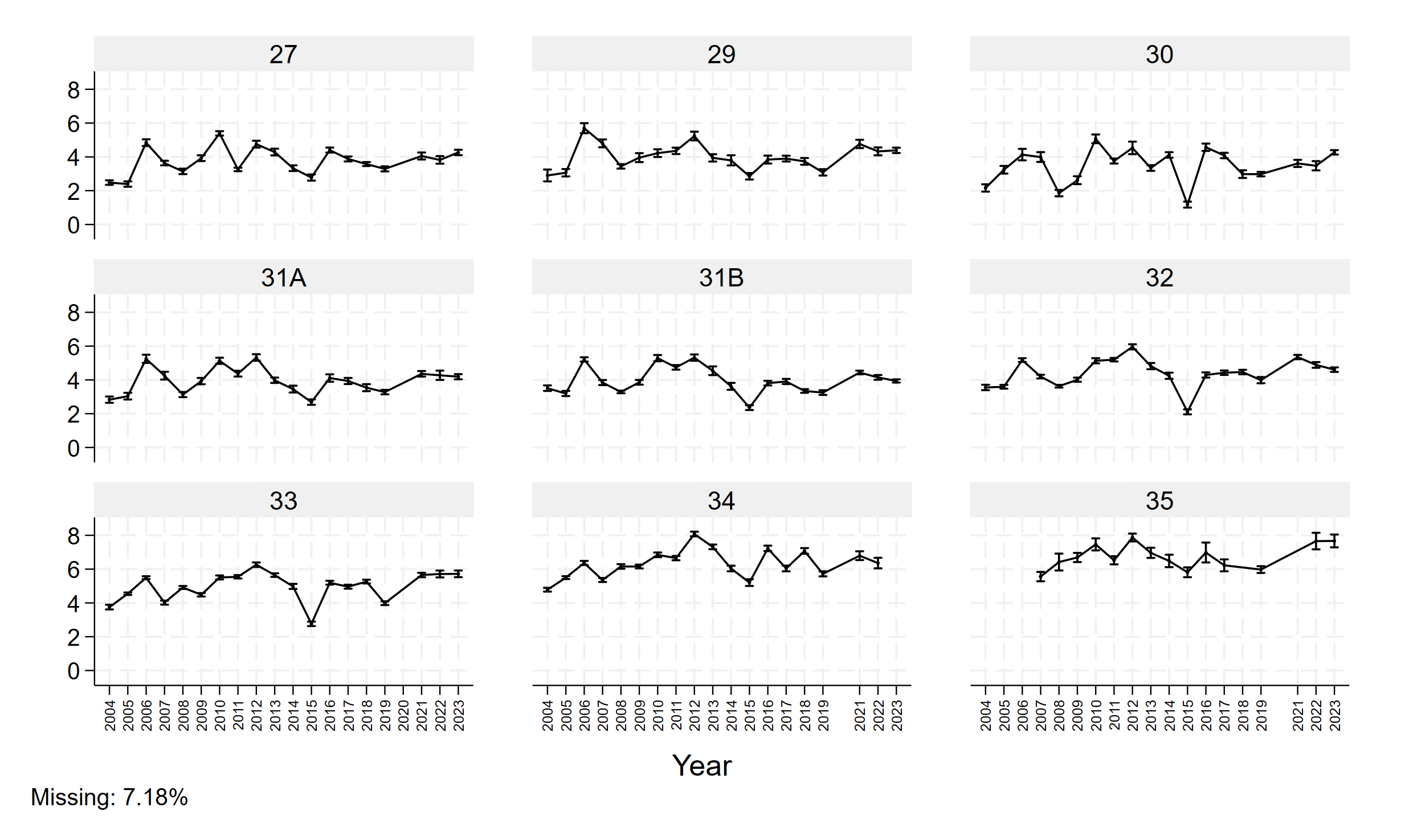


**Figure S6** Average May bottom water temperature (°C) with 95% confidence intervals by LFA from 2003 to 2023.

**Patterns of juvenile probability over different sampling months**

The estimated probability of sampling juvenile lobsters across different sampling months during the fishing seasons for each LFA is presented in Fig. S7. To account for annual and environmental variability, the predictor year was fixed at the most recent year of available data (2023), and water depth was held constant at eight meters across all LFAs. A group of LFAs with fishing seasons primarily from April to June (LFAs 29, 31A, 31B, and 32) exhibited similar patterns, with a slight increase in juvenile probability toward the end of the season. An exception was LFA 29, which displayed a relatively flat pattern; however, only two months of data were available for this area in 2023. LFAs 27 and 30, which span from May to July, also showed relatively flat trends across the three-month fishing period.

LFAs 33 and 34, which share an extended fishing season from November through June of the following year, demonstrated a modest increase in juvenile probability during the latter half of the season, with LFA 33 exhibiting a more pronounced upward trend. In contrast, LFA 35 had a distinct seasonal pattern, with a fall fishing season from October to December (although sampling in 2023 occurred only in October) and a spring season from April to July. During the spring, juvenile probability declined noticeably over time, with the lowest values observed in October. Overall, LFA 35 exhibited higher juvenile probabilities throughout its fishing seasons compared to the other LFAs.

**
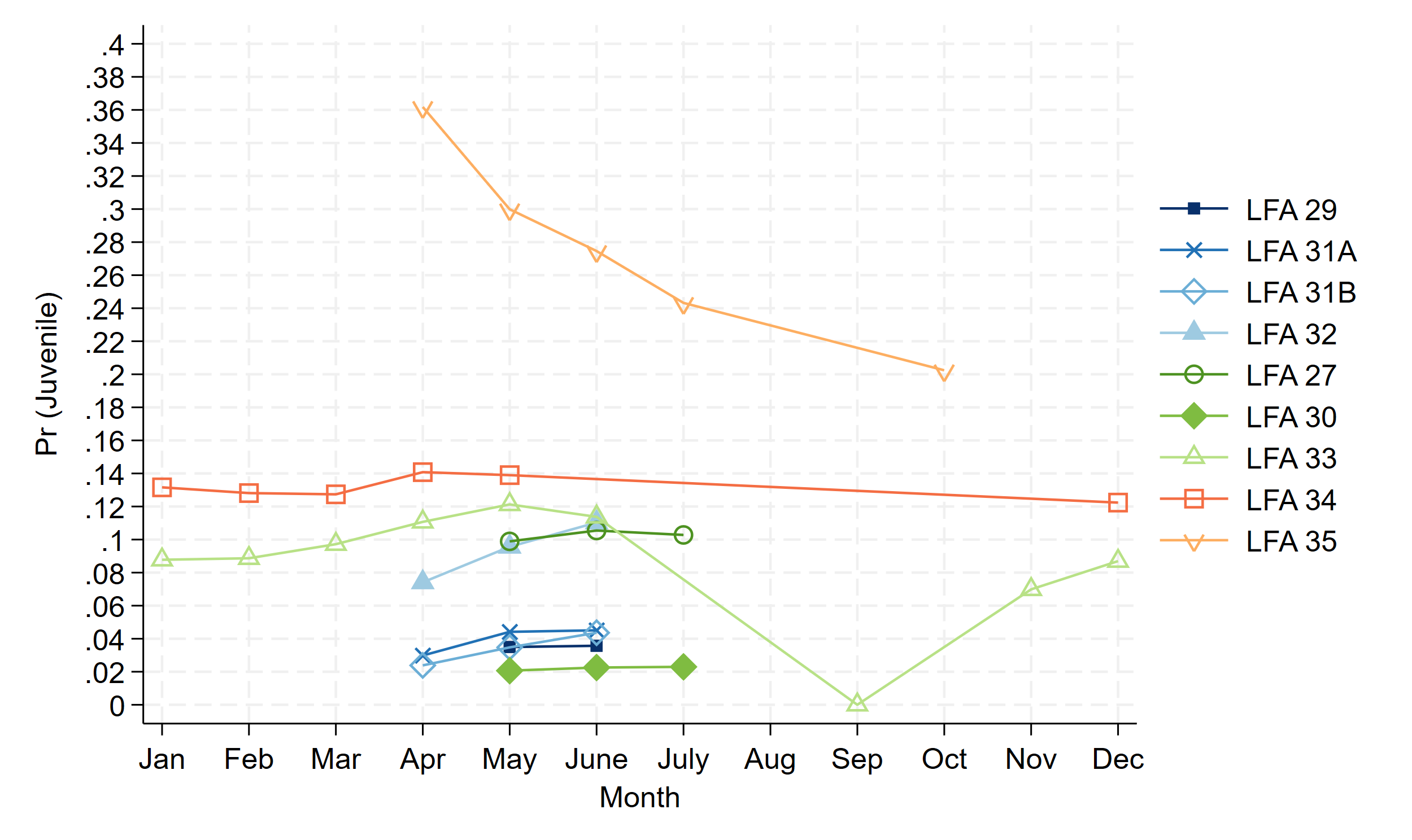
**

**Figure S7** Patterns of juvenile lobster probability (31–60 mm CL, representing a smaller size class) across sampling months for each LFA. Predictions were fixed at the year 2023 and water depth of eight meters for all LFAs.

**Patterns of juvenile probability influenced by LFA, water depth, and lobster sex**

The three-way interaction between LFA, water depth, and lobster sex on the probability of being juvenile lobsters is presented by plotting the LFA-Depth interaction separately for males and females (Fig. S8). To account for temporal effects, the year and month were fixed at 2023 and May, respectively. The estimated probability of sampling juvenile lobsters at varying water depths differed across LFAs. Most LFAs (31A, 27, 32, 33, 34, and 35) exhibited a decrease in juvenile probability with increasing depth, although the steepness of this decline varied between sexes. For example, LFA 34 exhibited a steeper decline in juvenile probability for females compared to males, whereas in LFA 27, the decline was more pronounced in males than in females.

In contrast, juvenile probability in LFA 30 increased with depth for both males and females. However, LFAs 29 and 31B exhibited contrasting trends between sexes, where juvenile probability gradually declined with depth for females but increased for males.

**
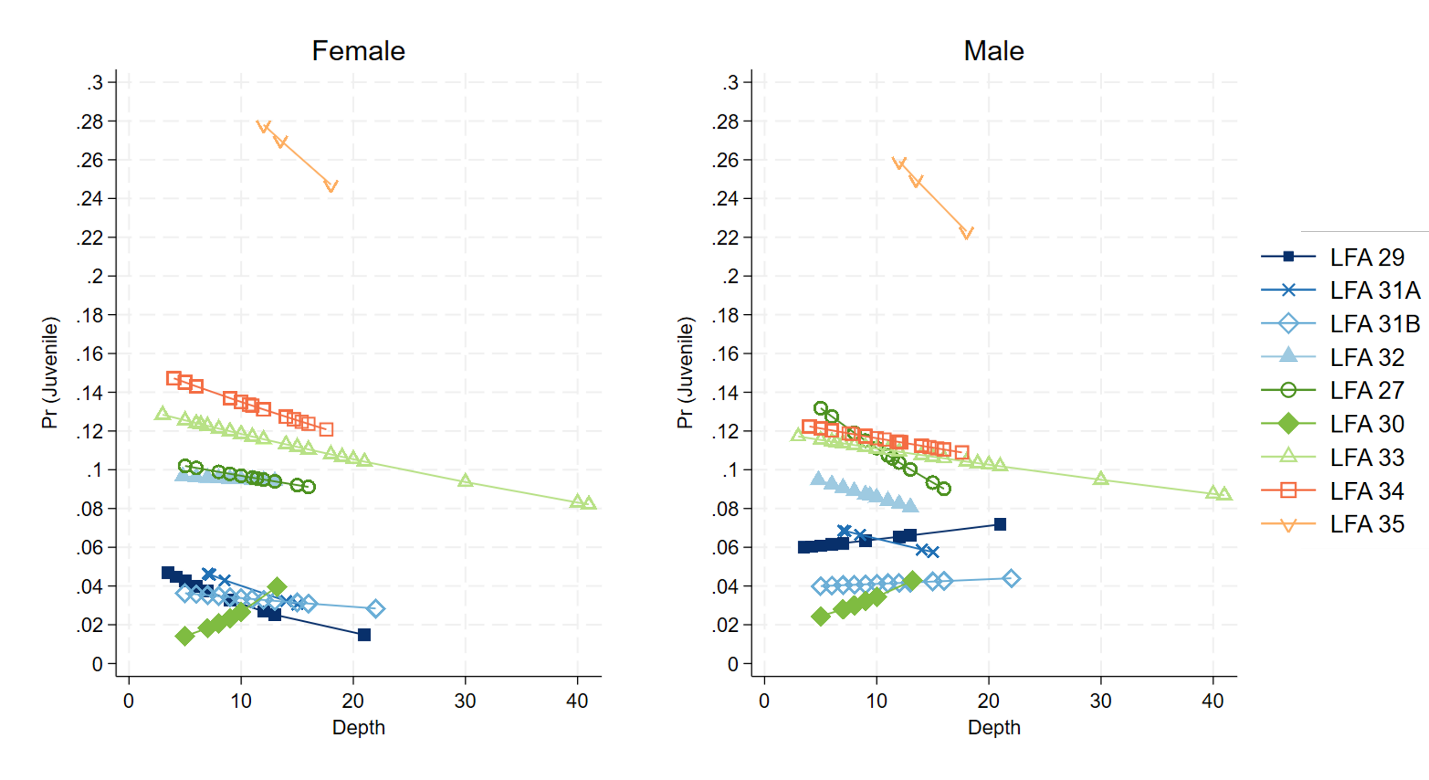
**

**Figure S8** Interaction between LFA and water depth on juvenile probability (31–60 mm CL, representing a smaller size class) for males and females. Predictions are based on the year fixed at 2023 and the month fixed at May.

**Table S4** Estimates of random effect parameters and intraclass correlation coefficients (ICCs) at each level from the mixed-effects linear and logistic regression models.

| Random effect parameter | Linear model |  | Logistic model | |  | |
| --- | --- | --- | --- | --- | --- | --- |
|  | Estimate (SE) | ICC | Estimate (SE) | | ICC | |
| Vessel code level | 17.191 (1.977) | 0.096 | 0.672 (0.089) | | 0.146 | |
| Segment-Vessel level | 5.963 (0.982) | 0.130 | 0.206 (0.048) | | 0.191 | |
| Segment level | 8.913 (0.734) | 0.180 | 0.321 (0.054) | | 0.261 | |
| Sampling event level | 4.444 (0.321) | 0.205 | 0.113 (0.035) | | 0.285 | |
| Lobster level | 141.673 (5.157) |  | 3.29 | |  | |
| Random slope parameter | Linear model | | Logistic model | | |  |
|  | Estimate (SE) | | Estimate (SE) | | |  |
| Day of Segment  (Regression coefficient) | -2.987 (0.269) |  | 0.322 (0.048) | |  |  |
| Segment (Variance) | 8.913 (0.734) |  | 0.321 (0.054) | |  |  |
| Day of Segment (Variance) | 61.937 (4.892) |  | 2.186 (0.375) | |  |  |
| Covariance | -14.773 (1.638) |  | -0.554 (0.130) | |  |  |
| Interpretation | | | | | |  |
| Approximate 95% range  of Day of Segment slopes | -$2.987\pm1.96\sqrt{61.937}$  (-18.412, 12.438) | | | 0.322$\pm1.96\sqrt{2.186}$  (-2.576, 3.220) | |  |
| Correlation between intercepts and slopes | -14.773/$\sqrt{8.913\times61.937}$  = -0.627 | | | -0.554/$\sqrt{0.321\times2.186}$  = -0.661 | |  |

The autocorrelations within a segment induced by the random slopes are quite small because only a small part of the unexplained variance resides at the segment level; the largest variability is between individual lobsters.
